# The oxygen level in air directs airway epithelial cell differentiation by controlling mitochondrial citrate export

**DOI:** 10.1126/sciadv.adr2282

**Published:** 2025-01-24

**Authors:** Bo Ram Kim, Adam J. Rauckhorst, Michael S. Chimenti, Tayyab Rehman, Henry L. Keen, Philip H. Karp, Eric B. Taylor, Michael J. Welsh

**Affiliations:** ^1^ Department of Internal Medicine, University of Iowa Carver College of Medicine, Iowa City, IA, USA.; ^2^ Pappajohn Biomedical Institute, University of Iowa Carver College of Medicine, Iowa City, IA, USA.; ^3^ Howard Hughes Medical Institute, University of Iowa, Iowa City, IA, USA.; ^4^ Department of Molecular Physiology and Biophysics, University of Iowa Carver College of Medicine, Iowa City, IA, USA.; ^5^ Fraternal Order of Eagles Diabetes Research Center, University of Iowa Carver College of Medicine, Iowa City, IA, USA.; ^6^ Iowa Institute of Human Genetics, University of Iowa Carver College of Medicine, Iowa City, IA, USA.

## Abstract

Oxygen controls most metazoan metabolism, yet in mammals, tissue O_2_ levels vary widely. While extensive research has explored cellular responses to hypoxia, understanding how cells respond to physiologically high O_2_ levels remains uncertain. To address this problem, we investigated respiratory epithelia as their contact with air exposes them to some of the highest O_2_ levels in the body. We asked how the O_2_ level in air controls differentiation of airway basal stem cells into the ciliated epithelial cells essential for clearing airborne pathogens from the lung. Through a metabolomics screen and ^13^C tracing on primary cultures of human airway basal cells, we found that the O_2_ level in air directs ciliated cell differentiation by increasing mitochondrial citrate export. Unexpectedly, disrupting mitochondrial citrate export elicited hypoxia transcriptional responses independently of HIF1α stabilization and at O_2_ levels that would be hyperoxic for most tissues. These findings identify mitochondrial citrate export as a cellular mechanism for responding to physiologically high O_2_ levels.

## Teaser

Mitochondria respond to high levels of oxygen in air and direct airway epithelia to produce ciliated cells that defend the lung.

## INTRODUCTION

Molecular oxygen (O_2_) is the most fundamental regulator of mammalian cellular metabolism. Despite its essentiality, physiological tissue O_2_ levels vary widely (*1*). Most investigations of how O_2_ levels control cellular function have focused on how low O_2_ levels contribute to pathophysiology, such as in cancer or ischemia. That research effort drove the foundational discovery that hypoxia engages the hypoxia-inducible factor 1α (HIF1α) pathway to control many cellular adaptations and functions (*2*–*4*).

The opposite end of the O_2_ spectrum is found at respiratory epithelia due to their contact with environmental air. The 21% O_2_ in air would be considered hyperoxic for most other tissues, which are exposed to 0.5 to 9% O_2_ (*1*). A previous report suggested that the level of O_2_ in air might be important because airway epithelia exposed to hypoxia had fewer ciliated cells (*5*). Ciliated airway epithelial cells provide a critical lung defense as their constantly beating cilia sweep mucus and inhaled pathogens out of the lung (*6*). Yet, the mechanisms by which the high O_2_ level in air controls ciliated cell differentiation from basal (stem) cells remain unknown, presenting an opportunity to address the basic problem of understanding how cells detect and signal physiologically high O_2_ levels.

Given oxygen’s master role in regulating cellular metabolism, we hypothesized that metabolic signaling may control ciliated cell differentiation. Through a metabolomics screen and deeper analysis using ^13^C tracing on human airway basal stem cells, we found that O_2_ levels in air direct the ciliated cell lineage decision by increasing mitochondrial citrate export. Moreover, disrupting mitochondrial citrate export induced aspects of the HIF1α transcriptional program independently of HIF1α stabilization. Discovery of a role for mitochondrial citrate export in the airway O_2_ response reveals a mechanism of mitochondrial signaling. This may also inform pathophysiology in respiratory epithelia.

## RESULTS

### Airway O_2_ tension regulates differentiation of human airway basal cells into ciliated cells

**Fig. 1.**
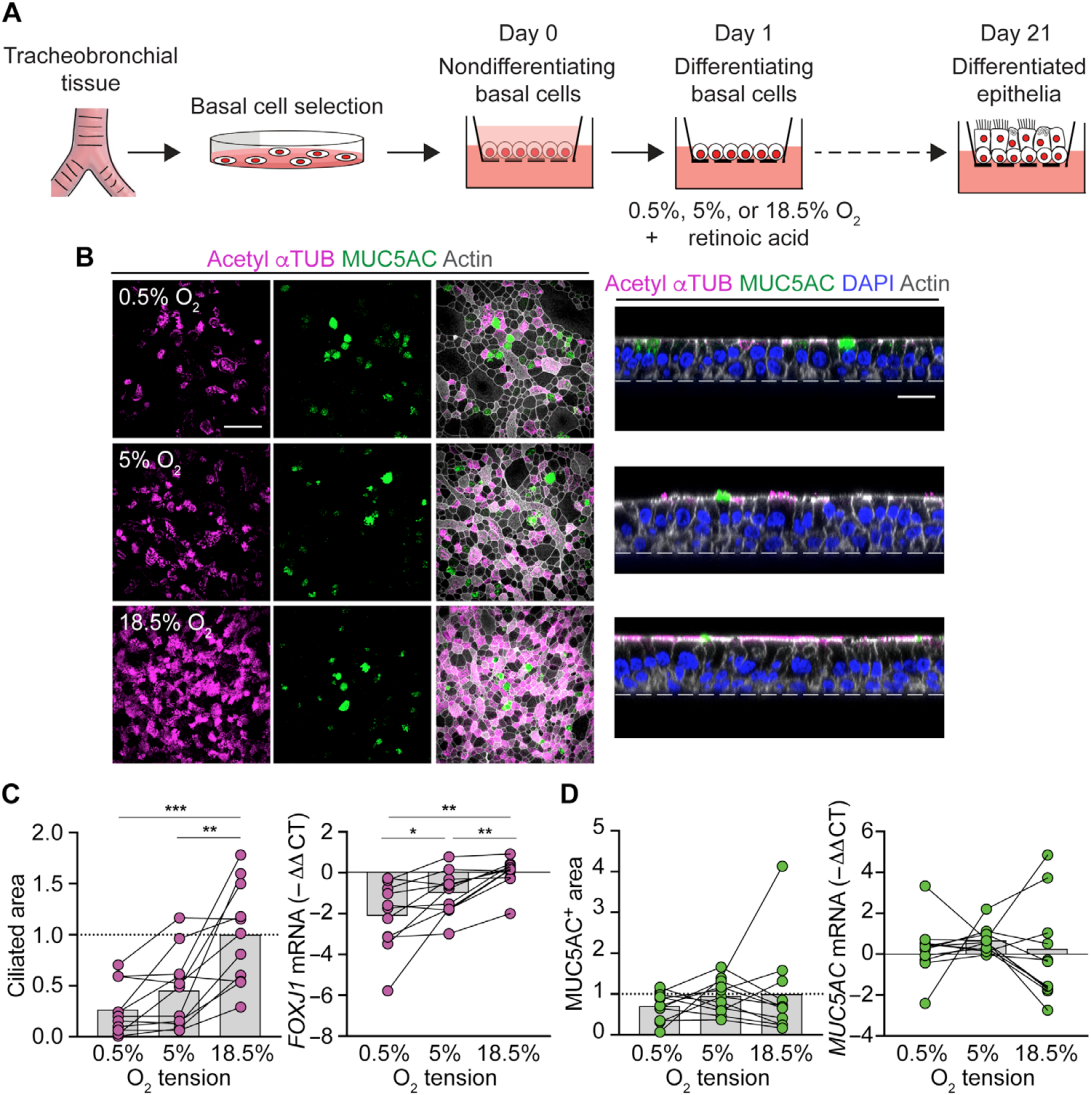
Airway O_2_ tension regulates differentiation of human airway basal cells into ciliated cells. (**A**) Schematic of in vitro differentiation of airway basal cells. (**B**) En face images of ciliated cells expressing acetyl-αTUB (magenta) and goblet cells expressing MUC5AC (green) in epithelia differentiated at 0.5, 5, and 18.5% O_2_. Cross section images are also shown. Scale bars, 50 μm (en face) and 20 μm (cross section). (**C**) % Ciliated area and real-time quantitative polymerase chain reaction (RT-qPCR) analysis of *FOXJ1* mRNA in epithelia differentiated at 0.5, 5, and 18.5% O_2_ (*n* = 11 donors). Data are normalized to 18.5% O_2_. (**D**) % MUC5AC-positive area and RT-qPCR analysis of *MUC5AC* mRNA in epithelia differentiated at 0.5, 5, and 18.5% O_2_ (*n* = 11 donors). Data are normalized to 18.5% O_2_. Bars represent mean. *P < 0.05, **P < 0.01, and ***P < 0.001 using repeated measures analysis of variance (ANOVA) with Tukey’s multiple comparisons test [(C) and (D)].

We aimed to mechanistically decipher the unexplained role of O_2_ tension in differentiation of airway basal cells into ciliated cells. We isolated human airway basal cells from donor tracheal or bronchial tissue and induced their differentiation by apical exposure to an air-liquid interface (ALI) (*7*). We varied the O_2_ tension at the ALI to 18.5, 5, or 0.5% O_2_ to model normoxic airway O_2_ tension, normoxic O_2_ tension for most other tissues, and hypoxic O_2_ tension, respectively (*1*). After 18 to 21 days, we tracked differentiation into ciliated cells (Fig. 1A). Compared to both 5 and 0.5% O_2_, 18.5% O_2_ markedly increased the number of ciliated cells, assessed by acetyl–α-tubulin (αTUB)–or β-tubulin (βTUB) IV–positive area and mRNA levels for a major ciliated cell transcription factor FOXJ1 (*8*–*10*) (Fig. 1, B and C). Conversely, O_2_ tension did not significantly affect differentiation into goblet cells, assessed by mRNA levels and area positive for a major goblet cell mucin MUC5AC (Fig. 1, B and D) (*11*). To evaluate whether decreased differentiation into ciliated cells at low O_2_ tensions is due to cytotoxicity, we measured transepithelial ion transport, an important function of healthy epithelia. Epithelia formed under different O_2_ tensions had typical short circuit current and transepithelial conductance responses, suggesting an intact cellular function (fig. S1, A and B). Together, these data indicate that physiological airway O_2_ tension is necessary for differentiation into ciliated but not goblet cells.

### Airway O_2_ tension activates mitochondrial respiration in differentiating basal cells

**Fig. 2.**
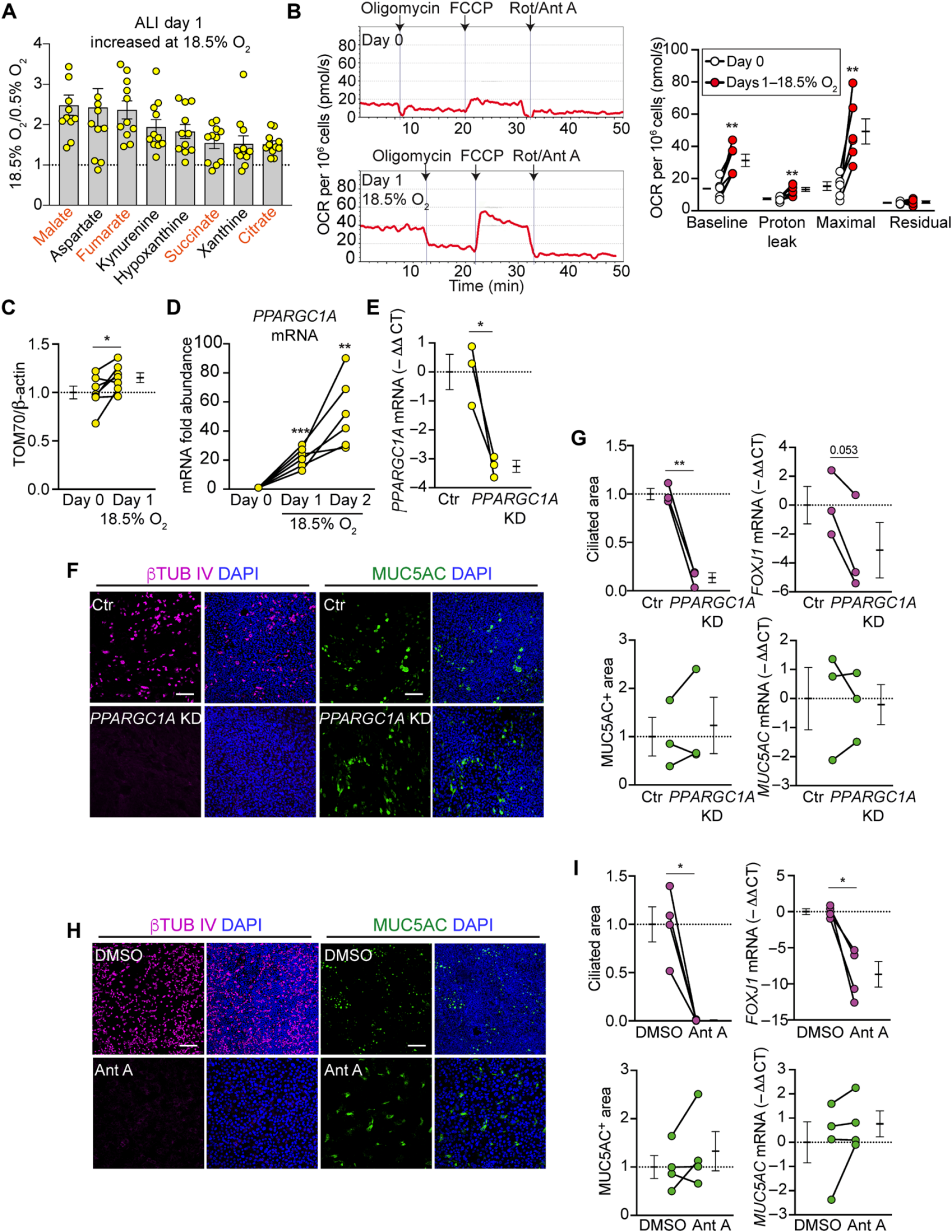
Airway O_2_ tension activates mitochondrial respiration in differentiating basal cells. (**A**) Top eight significantly increased metabolites at 18.5% O_2_. TCA cycle metabolites are highlighted in orange (*n* = 11 donors). (**B**) Traces of oxygen consumption rate (OCR) in nondifferentiating basal cells at day 0 and basal cells differentiated for 1 day at 18.5% O_2_ (left) and quantification of OCR measurements (right). Rot, rotenone; Ant A, antimycin A (*n* = 6 donors). (**C**) TOM70 protein quantification normalized to β-actin (*n* = 7 donors). (**D**) RT-qPCR analysis of *PPARGC1A* mRNA induction at 18.5% O_2_. Data are normalized to mRNA at day 0 (*n* = 6 donors). (**E** to **G**) Basal cells were transduced with shLuciferase or shPPARGC1A-carrying lentivirus and differentiated at an ALI. (E) RT-qPCR analysis of *PPARGC1A* mRNA at ALI day 2. Ctr: shLuciferase control. KD: *PPARGC1A* knockdown by shPPARGC1A (*n* = 3 donors). (F) En face images of ciliated cells expressing βTUB IV (magenta) and goblet cells expressing MUC5AC (green). Scale bars, 100 μm. (G) % Ciliated and MUC5AC-positive areas and RT-qPCR analysis of *FOXJ1* and *MUC5AC* mRNA. Data are normalized to shLuciferase-transduced control (*n* = 3 donors). (**H** and **I**) Basal cells were treated with either dimethyl sulfoxide (DMSO) (vehicle control) or 2 μM antimycin A during differentiation at an ALI. (H) En face images of ciliated cells expressing βTUB IV (magenta) and goblet cells expressing MUC5AC (green). Scale bars, 100 μm. (I) % Ciliated and MUC5AC-positive area and RT-qPCR analysis of *FOXJ1* and *MUC5AC* mRNA. Data are normalized to DMSO control (*n* = 4 donors). Data represent means ± SEM. *P < 0.05, **P < 0.01, and ***P < 0.001 using repeated measures ANOVA with Dunnett’s multiple comparisons test (A), paired Student’s *t* test [(B), (C), (E), (G), and (I)] and one sample *t* test (D).

Given the intrinsic relationship between oxygenation and metabolism, we performed a metabolomics screen to identify mechanisms that may regulate differentiation into ciliated cells. To capture changes during the initial phases of the ciliated cell linage decision, we analyzed basal cells differentiated for 1 day either at 0.5 or 18.5% O_2_. Strikingly, four of the top eight significantly elevated metabolites at 18.5% versus 0.5% O_2_ were tricarboxylic acid (TCA) cycle metabolites (Fig. 2A and data S1).

That result led us to test mitochondrial oxidative phosphorylation by measuring O_2_ consumption at days 0 and 1 of differentiation at 18.5% O_2_. Differentiation caused basal- and carbonyl cyanide-*p*-trifluoromethoxyphenylhydrazone (FCCP)–stimulated maximal mitochondrial O_2_ consumption rates to double (Fig. 2B). Because increased TCA cycle metabolites and O_2_ consumption suggested an increase in mitochondrial content and function, we tested that idea with additional experiments. We found that 18.5% O_2_ increased the abundance of the mitochondrial outer membrane protein TOM70 (Fig. 2C). 18.5% O_2_ also increased *PPARGC1A* mRNA, which encodes PGC1α, a master transcriptional regulator of mitochondrial biogenesis (Fig. 2D and fig. S2) (*12*). Knocking down *PPARGC1A* suppressed ciliated cell but not goblet cell differentiation (Fig. 2, E to G). Moreover, inhibiting mitochondrial respiration with antimycin A prevented basal cells from differentiating into ciliated cells but not into MUC5AC-positive cells (Fig. 2, H and I). These data suggest that 18.5% O_2_ increases mitochondrial amount and respiration preceding ciliated cell development and is required for differentiation into ciliated cells.

Many mitochondria-derived metabolites can function as signaling molecules (*13*, *14*). Hypoxia increases 2-hydroxygluterate (2HG) (*15*), and we found that 0.5% O_2_ increased 2HG in differentiating basal cells (data S1). 2HG has two enantiomers, L- and R-2HG, and both enantiomers regulate gene expression by inhibiting many histone, RNA, and DNA demethylases (*16*, *17*). Therefore, we asked whether 2HG could inhibit ciliated cell differentiation. Adding the cell-permeable octylated forms of L-2HG and R-2HG increased 2HG levels in basal cells cultured at 18.5% O_2_ (fig. S3A). However, the enantiomers decreased both ciliated cell and goblet cell differentiation (fig. S3, B and C). These data suggest that 2HG may inhibit general differentiation rather than specifically inhibiting ciliated cell differentiation.

### Airway O_2_ tension promotes mitochondrial citrate export in differentiating basal cells

**Fig. 3.**
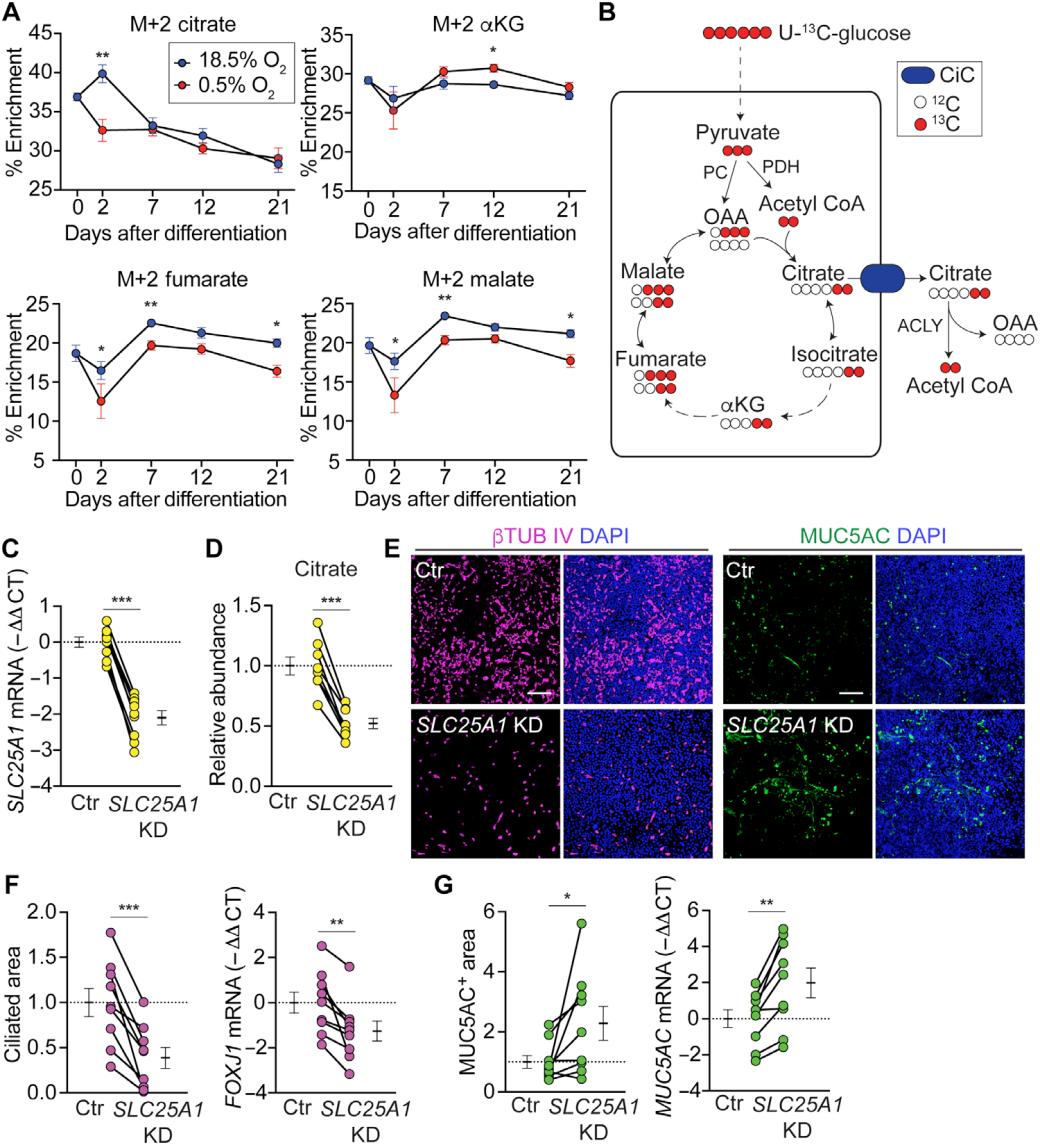
Mitochondrial citrate export is necessary for the ciliated cell lineage decision. (**A**) % M+2 ^13^C isotopolog of major TCA cycle metabolites during differentiation at 18.5% O_2_ (blue circle) and 0.5% O_2_ (red circle) (*n* = 6 donors). (**B**) Schematic illustration of how different isotopologues are formed from [U]-^13^C-glucose. Dashed line indicates multiple steps. αKG, alpha-ketoglutarate; OAA, oxaloacetate; PC, pyruvate carboxylase; PDH, pyruvate dehydrogenase. (**C** to **G**) Basal cells were transduced with either shScramble control or shSLC25A1-lentivirus and differentiated at an ALI. (C) RT-qPCR analysis showing knockdown of *SLC25A1* mRNA in shSLC25A1-transduced cells at ALI day 2 (*n* = 9 donors). (D) Relative abundance of citrate in control (Ctr) and *SLC25A1* knockdown (KD) cells at ALI day 2 (*n* = 6 donors, two donors were used twice in independent experiments). (E) En face images of ciliated cells expressing βTUB IV (magenta) and goblet cells expressing MUC5AC (green). Scale bars, 100 μm. (F) % Ciliated area and RT-qPCR analysis of *FOXJ1* mRNA in differentiated epithelia. Data are normalized to shScramble-transduced control (*n* = 9 donors). (G) % MUC5AC-positive area and RT-qPCR analysis of *MUC5AC* mRNA in differentiated epithelia. Data are normalized to shScramble-transduced control (*n* = 9 donors). Data represent means ± SEM. *P < 0.05, **P < 0.01, and ***P < 0.001 using paired Student’s *t* test [(A), (C), (D), (F), and (G)].

To gain deeper insight into how mitochondrial respiration might regulate differentiation into ciliated cells, we traced uniformly labeled [U]-^13^C-glucose into TCA cycle metabolites at differentiation days 0, 2, 7, 12, and 21 (fig. S4 and data S2). Intriguingly, 18.5% O_2_ but not 0.5% O_2_ increased abundance of the citrate M+2 isotopolog at day 2 (Fig. 3A). Among TCA cycle metabolites, this increase was unique to citrate. In contrast, the M+3 isotopolog showed similar labelling patterns between O_2_ tensions for all major TCA cycle intermediates (fig. S5). Because it reflects increased forward, oxidative TCA cycle fluxes through pyruvate dehydrogenase versus reverse fluxes through pyruvate carboxylase, this pattern of increased M+2 but not M+3 citrate is consistent with increased glucose oxidation at a higher O_2_ tension. The peculiar discordance in the M+2 labelling pattern between citrate and other TCA cycle intermediates could arise if mitochondrial citrate export increased (Fig. 3B). These data led us to speculate that citrate exiting the mitochondria may cue the ciliated cell lineage decision.

### Mitochondrial citrate export is necessary for the ciliated cell lineage decision

**Fig. 4.**
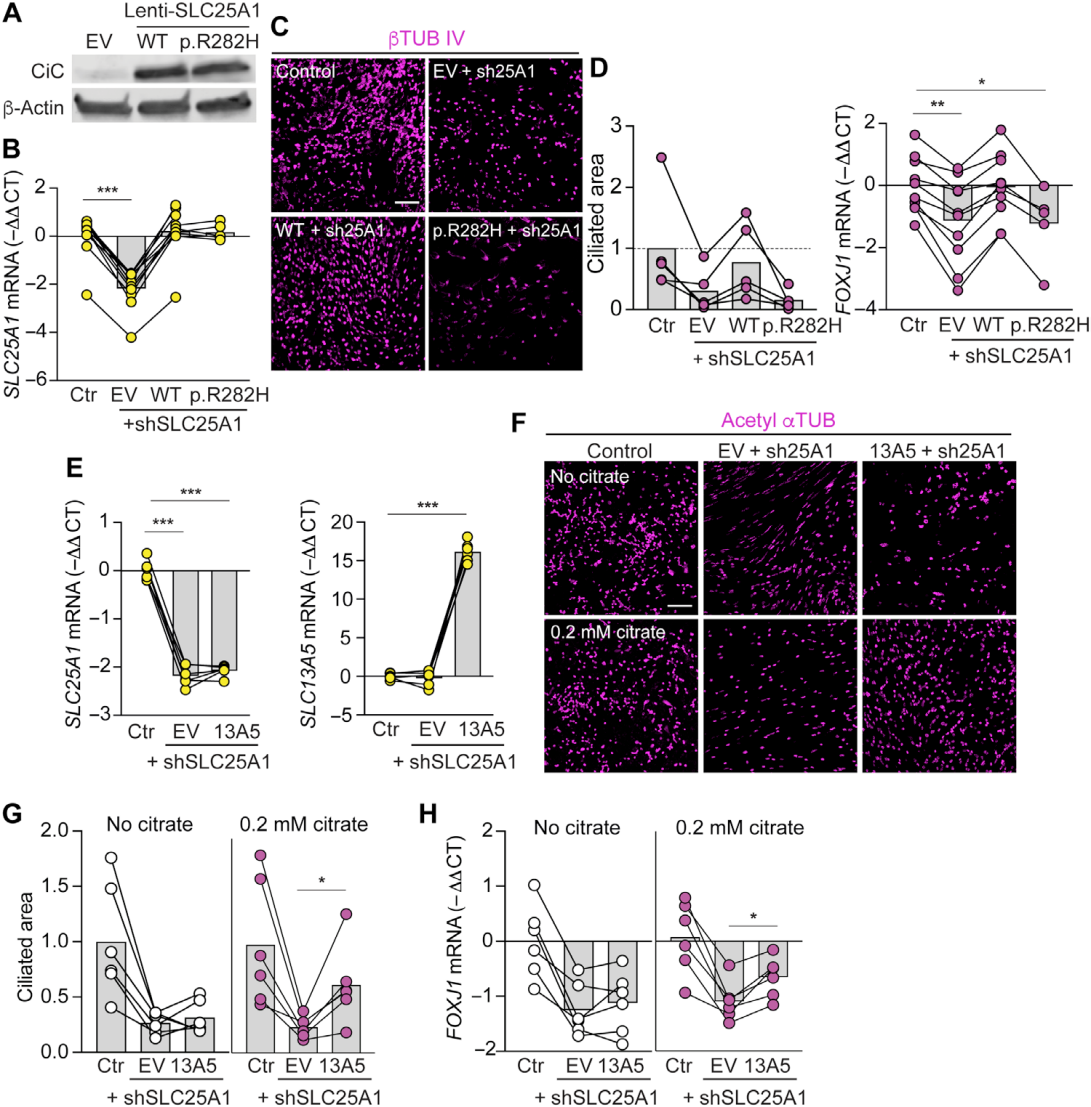
Wild type but not transport-dead CiC rescues ciliated cell differentiation. (**A**) Basal cells were transduced with lenti–empty vector (EV), lenti-wild type (WT), or lenti-p.R282H-mutant CiC. Western blot showing similar levels of WT and p.R282H-mutant CiC protein over endogenous levels in EV-transduced cells. (**B** to **D**) Control group (Ctr) was co-infected with shScramble and lenti-EV viruses. Other groups were co-infected with shSLC25A1 and lenti-EV or lenti-WT CiC or lenti-p.R282H-mutant CiC viruses. Transduced basal cells were differentiated at an ALI. (B) RT-qPCR analysis of *SLC25A1* mRNA at ALI day 2. Data are normalized to expression in the control group (*n* = 10 donors except for the p.R282H + shSLC25A1 group, which has *n* = 5 donors). (C) En face images of ciliated cells expressing βTUB IV. Scale bar, 100 μm. (D) % Ciliated area and RT-qPCR analysis of *FOXJ1* mRNA in differentiated epithelia. Data are normalized to the control group (ciliated area, *n* = 5 donors; RT-qPCR, *n* = 10 donors except for the p.R282H + shSLC25A1 group, which has *n* = 5 donors). (**E** to **H**) Control group (Ctr) was co-infected with shScramble and lenti–empty vector viruses. Other groups were co-infected with shSLC25A1 and lenti-EV or lenti-SLC13A5 viruses. Transduced basal cells were differentiated at an ALI. (E) RT-qPCR analysis of *SLC25A1* and *SLC13A5* mRNA at ALI day 2. Data are normalized to expression in the control group (n = 6 donors). (F) En face images of ciliated cells expressing acetyl-αTUB. Scale bar, 100 μm. [(G) and (H)] % Ciliated area (G) and RT-qPCR analysis of *FOXJ1* mRNA (H). Data are normalized to the control group (*n* = 6 donors). Bars represent mean. *P < 0.05, **P < 0.01, and ***P < 0.001 using mixed-effects analysis with Dunnett’s multiple comparisons test [(B) and (D)], repeated measures ANOVA with Dunnett’s multiple comparisons test (E), and with Tukey’s multiple comparisons test [(G) and (H)].

To test whether mitochondrial citrate export gates differentiation into ciliated cells, we knocked down the mitochondrial citrate carrier (CiC) encoded by the *SLC25A1* gene (Fig. 3C) (*18*). *SLC25A1* knockdown decreased both citrate abundance and differentiation into ciliated cells (Fig. 3, D to G), indicating that at 18.5% O_2_, CiC is required for maintaining cellular citrate levels and differentiation into ciliated cells. We next aimed to specifically evaluate citrate transport versus a structural or other nontransport role of the CiC. We complemented *SLC25A1* knockdown by ectopically expressing either wild-type or a transport-dead p.R282H CiC mutant identified in human patients with congenital myasthenic syndromes (*19*, *20*). Protein levels of wild type and the p.R282H mutant were similar (Fig. 4A), corroborating the previous report that p.R282H is not destabilizing (*20*). Wild-type but not p.R282H mutant CiC rescued differentiation into ciliated cells (Fig. 4, B to D), suggesting that citrate transport is indeed the CiC function that modulates differentiation.

To further test the role of citrate in differentiation into ciliated cells, we supplemented citrate in the culture media to increase cellular citrate levels independently of CiC function. At physiologic pH, citrate is a trivalent anion requiring the plasma membrane sodium-dependent citrate transporter encoded by *SLC13A5* to enter the cell (*21*). Native *SLC13A5* expression was barely detectable in airway basal cells (fig. S6A), suggesting that mitochondria are the major source of intracellular citrate. We ectopically expressed *SLC13A5* to facilitate uptake of supplemented citrate and confirmed increased intracellular citrate levels (fig. S6B). Levels of other TCA metabolites also increased, indicating that the supplemented citrate infiltrated cellular metabolism. We next tested the effects of combined *SLC13A5* complementation and citrate supplementation on differentiation of *SLC25A1* knockdown basal cells. Ectopic *SLC13A5* expression with, but not without, citrate supplementation partially rescued differentiation of *SLC25A1* knockdown basal cells into ciliated cells (Fig. 4, E to H), demonstrating that the citrate molecule regulates differentiation. The observed partial versus complete rescue may result from differing compartmentalization of mitochondrially exported and extracellularly imported citrate (*22*).

### Mitochondrial citrate export promotes the ciliated cell lineage decision via ATP citrate lyase

**Fig. 5.**
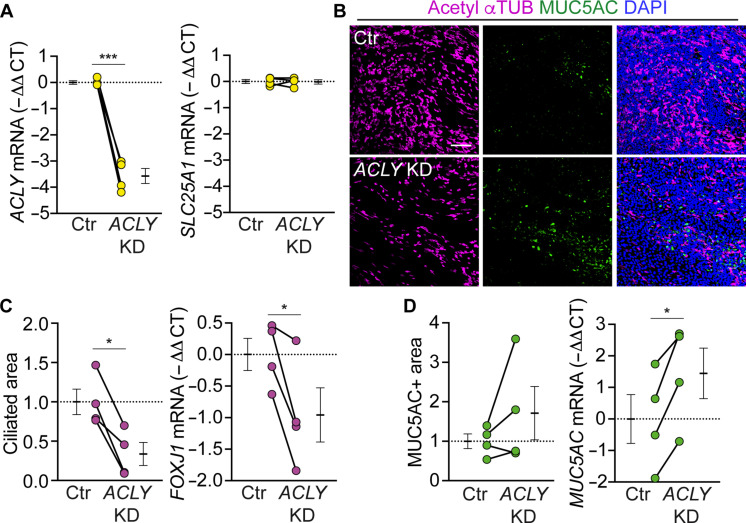
Citrate export promotes the ciliated cell lineage decision via ATP citrate lyase. (**A**) RT-qPCR analysis of *ACLY* and *SLC25A1* mRNA in shACLY-transduced cells at ALI day 2. Data are normalized to expression in shScramble-transduced control (Ctr) (*n* = 4 donors). (**B**) En face images of ciliated cells expressing acetyl-αTUB (magenta) and goblet cells expressing MUC5AC (green). Scale bar, 100 μm. (**C**) % Ciliated area and RT-qPCR analysis of *FOXJ1* mRNA. Data are normalized to expression in shScramble-transduced control (*n* = 4 donors). (**D**) % MUC5AC-positive area and RT-qPCR analysis of *MUC5AC* mRNA. Data are normalized to shScramble-transduced control (*n* = 4 donors). Data represent means ± SEM. *P < 0.05 and ***P < 0.001 using paired Student’s *t* test [(A), (C), and (D)].

We then asked how mitochondrial citrate export might regulate gene expression directing the ciliated cell lineage decision. Lineage commitment in stem cells is governed by the coordinated action of lineage-specific transcription factors and chromatin remodelers and modifiers, which drive epigenetic changes specifying lineage choice (*13*). Cytosolic citrate, when cleaved by adenosine 5′-triphosphate (ATP) citrate lyase (ACLY), generates acetyl-CoA, an essential substrate for histone acetyltransferases (HATs) (*23*). Previous studies have shown that ACLY controls differentiation of other tissue stem cells (*23*–*26*). To test whether ACLY mediates transcriptional activation of ciliated cell lineage genes downstream of citrate export, we suppressed ACLY activity by short hairpin RNA (shRNA)–mediated knockdown or with the chemical inhibitor BMS-303141. *ACLY* knockdown phenocopied *SLC25A1* knockdown (Fig. 5, A to D). BMS-303141 treatment also decreased ciliated cell differentiation in a concentration-dependent manner (fig. S7, A to C). These data are consistent with citrate export promoting the ciliated cell lineage decision via ACLY. Acetate is another major source of acetyl-CoA for HATs through the action of acyl-CoA synthetase short-chain family member 2 (ACSS2) (*22*). As an additional test of acetyl-CoA-directed ciliated cell differentiation, we ectopically expressed ACSS2 and supplemented media with 2 mM acetate (in addition to the 1.8 mM in media). ACSS2 expression increased acetyl-CoA levels, with no effect of additional acetate, suggesting that 1.8 mM acetate is saturating (fig. S8, A and B). However, ACSS2 and acetate did not rescue and instead further decreased ciliated cell differentiation in *SLC25A1* knockdown basal cells (fig. S8, C to E). These results suggest that acetyl-CoA produced by ACSS2 from acetate and acetyl-CoA produced by ACLY are functionally distinct in airway basal cells as previously observed in neurons (*27*).

### Citrate export regulates chromatin accessibility and transcriptional activation of ciliated cell lineage genes

**Fig. 6.**
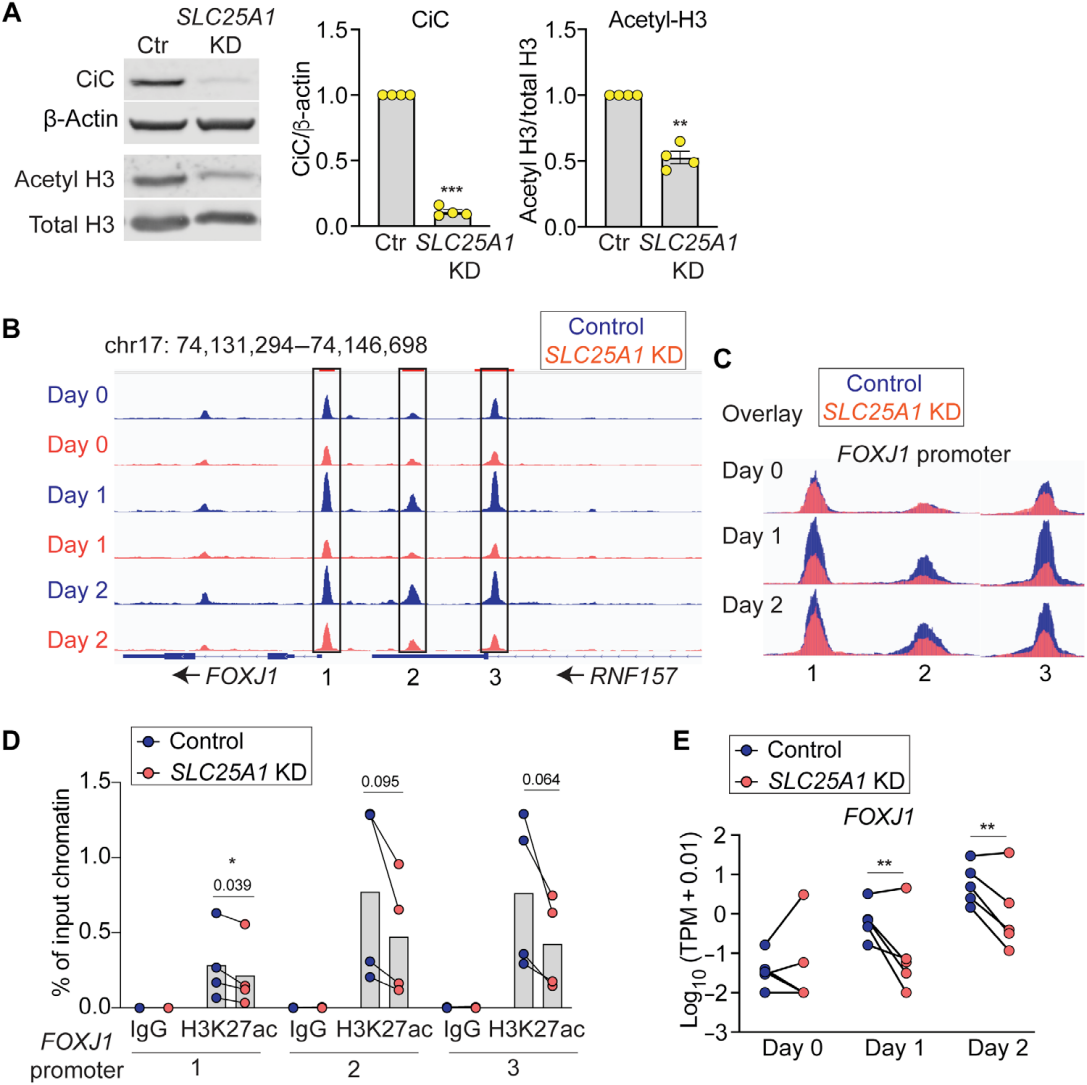
*SLC25A1* knockdown decreases chromatin accessibility and mRNA expression of the *FOXJ1* gene. (**A**) Western blot showing decreased acetylated histone 3 (H3) levels in *SLC25A1* knockdown (KD) cells at ALI day 2 compared to shScramble-transduced control. Acetylated H3 protein was blotted using an antibody that detects acetyl K9, K14, K18, K23, and K27. The graphs show quantification of CiC protein normalized to βActin (left), and acetyl H3 normalized total H3 (right) (*n* = 4 donors). (**B**) Normalized ATAC-seq sequencing tracks showing the promoter region of the *FOXJ1* gene at day 0 (predifferentiation), day 1, and day 2 post-differentiation. Each peak represents the average of five donors. Differentially accessible regions (*P* < 0.05) between control and knockdown cells at day 1 and/or day 2 are shown in box. Arrows point to the direction of transcription. (**C**) Overlay of differentially accessible ATAC-seq peaks shown in (B). (**D**) Chromatin Immunoprecipitation (ChIP) of acetylated K27 at histone 3 (H3K27ac) in control and *SLC25A1* KD cells at ALI day 2. The enrichment of H3K27ac in the 3 *FOXJ1* promoter regions that were less accessible in CiC KD cells are measured (*n* = 4 donors). (**E**) RNA-seq expression values for *FOXJ1* in log10 (TPM + 0.01) (*n* = 5 donors). Statistical significance was calculated by DESeq2 using the Wald test. Bars represent means ± SEM (A). **P < 0.01 and ***P < 0.001 using one sample *t* test (A) and paired Student’s *t* test (D).

Given the role of ACLY in providing the acetyl group for HATs, we hypothesized that mitochondrial citrate export affects histone acetylation. *SLC25A1* knockdown decreased global histone H3 acetylation levels by ~50% in differentiating basal cells (Fig. 6A). To test the role of CiC in regulating chromatin accessibility and mRNA transcripts for the ciliated cell lineage decision, we performed combined Assay for Transposase-Accessible Chromatin using sequencing (ATAC-seq) and RNA sequencing (RNA-seq) at day 0, day 1, and day 2 after differentiation with and without *SLC25A1* knockdown.

*SLC25A1* knockdown altered chromatin accessibility and mRNA expression of genes known to regulate differentiation. *FOXJ1* encodes a master transcription factor driving ciliated cell differentiation. *Foxj1*-null mice lack airway ciliated cells, and *Foxj1* overexpression causes ectopic ciliation in alveolar spaces (*8*–*10*). The *FOXJ1* promoter harbored three ATAC-seq peaks that were all less accessible in *SLC25A1* knockdown cells (Fig. 6, B and C). To directly test for decreased histone acetylation at the *FOXJ1* locus, we performed ChIP against acetylated H3K27 (H3K27ac) and compared its enrichment in the three *FOXJ1* promoter regions that were less accessible in *SLC25A1* knockdown cells. *SLC25A1* knockdown decreased H3K27ac in all three *FOXJ1* promoter regions (Fig. 6D). *SLC25A1* knockdown also decreased *FOXJ1* mRNA (Fig. 6E), strongly suggesting that citrate-mediated chromatin accessibility regulates *FOXJ1* gene transcription.

We also observed decreased chromatin accessibility in the promoter and/or potential enhancer elements and decreased mRNA expression of several other genes necessary for ciliated cell differentiation (*GMNC*, *MCIDAS*, *RFX2*, and *MYB*) (fig. S9, A and C) (*28*–*31*). Notch signaling antagonizes ciliated cell differentiation (*32*–*34*). In contrast to the pro-ciliated cell lineage genes, chromatin regions of *NOTCH2* and its ligand *JAG1* were more open in *SLC25A1* knockdown cells (fig. S9, B and D). *JAG1* mRNA levels were also higher in *SLC25A1* knockdown cells (fig. S9B), suggesting increased Notch signaling. Together, these data support a key role of mitochondrial citrate export in activating transcriptional networks driving the ciliated cell lineage decision.

### Blocking citrate export induces similar transcriptional patterns as does hypoxia without inducing HIF1α

**Fig. 7.**
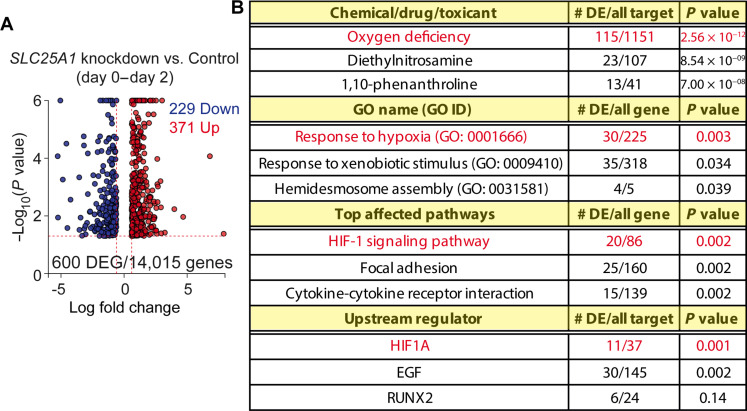
Blocking citrate export induces similar transcriptional patterns as does hypoxia. (**A** and **B**) Differentially expressed genes (DEGs) across day 0 to day 2 were identified using a likelihood ratio test with the day and condition (control versus *SLC25A1* knockdown) interaction term. The resulting gene sets were analyzed using Advaita Bio’s iPathwayGuide. (A) Volcano plot: 600 DEGs are shown. The dotted lines represent the thresholds for DEG selection: 0.6 for expression change and 0.05 for significance. (B) From top to bottom: Top three upstream chemicals, drugs, toxicants predicted as present (or overly abundant). Top three Gene Ontology (GO) terms of biological processes identified using the high-specificity pruning method. Top three affected pathways scored using the impact analysis method. Top three upstream regulators predicted as activated.

To better understand the functional role of mitochondrial citrate export, we screened for signaling pathways perturbed by *SLC25A1* knockdown. We analyzed our RNA-seq data with a model conditioned on the interaction of “day” and “knockdown status” (control versus *SLC25A1* knockdown). Using a likelihood ratio test, we identified 600 statistically significant genes that behaved differently across time points (day 0 to day 2) depending on *SLC25A1* expression (Fig. 7A). Pathway analysis was performed using iPathwayGuide software tool, which incorporates both pathway topology and enrichment in identifying affected pathways (*35*, *36*). Results converged upon hypoxia-related pathways as most strongly induced by *SLC25A1* knockdown during differentiation (Fig. 7B). CiC disruption perturbed many of the same transcripts as “oxygen deficiency.” Gene Ontology (GO) enrichment analysis also revealed “response to hypoxia” (GO:0001666) as the most enriched GO term of biological process. Consistent with the theme of hypoxia, the HIF1 signaling pathway was the most affected pathway. Moreover, HIF1α was the top upstream regulator predicted to be activated, indicating that *SLC25A1* knockdown induced many of the same target genes as HIF1α. This was especially unexpected given that these cells were differentiating at 18.5% O_2_, far from the hypoxic range. These results suggested a role for CiC in signaling the O_2_ level, a role for HIF1α in differentiation into ciliated cells, or both.

**Fig. 8.**
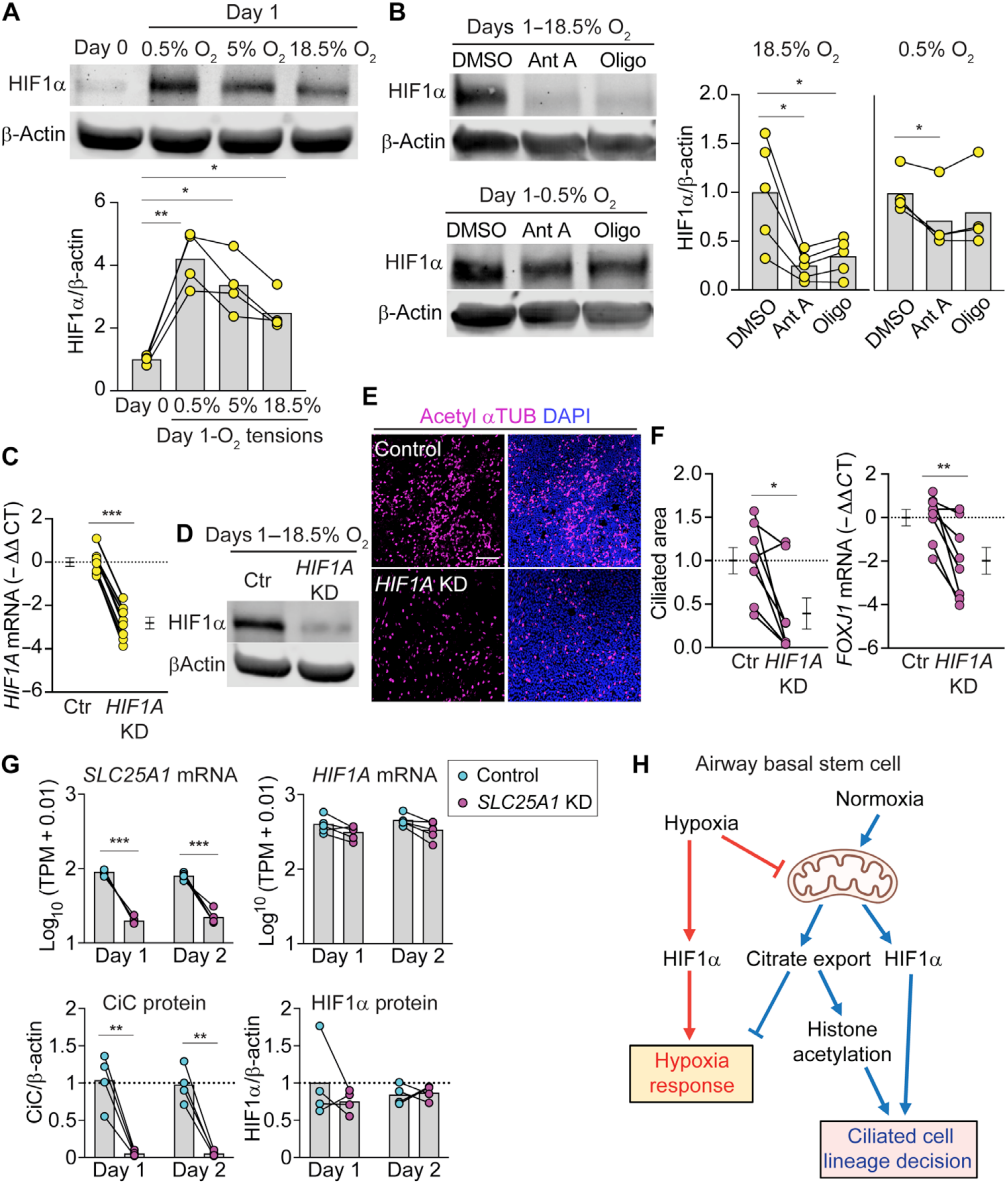
HIF1α stabilization in normoxia by mitochondrial respiration is required for differentiation into ciliated cells. (**A**) Western blot of HIF1α protein in nondifferentiating basal cells (day 0) and basal cells differentiated for 1 day at 0.5, 5, and 18.5% O_2_. Graph shows quantification of HIF1α protein normalized to β-actin (*n* = 4 donors). (**B**) Western blot of HIF1α protein in basal cells differentiated for 1 day at 0.5 and 18.5% O_2_. Cells were treated overnight with DMSO vehicle or 1 μM antimycin A or 1 μM oligomycin (Oligo). Graph shows HIF1α protein quantification normalized to β-actin (*n* = 4 donors for 0.5% O_2_; *n* = 5 for 18.5% O_2_). (**C** to **F**) Basal cells transduced with either shScramble control or shHIF1A lentivirus. Transduced cells were differentiated at 18.5% O_2_. Two different hairpins for *HIFIA* were used in independent experiments and results are combined. (C) RT-qPCR analysis of *HIF1A* mRNA at ALI day 2 (*n* = 8). (D) Western blot showing HIF1α KD. (E) En face images of ciliated cells expressing βTUB IV (magenta). Scale bar, 100 μm. (F) % Ciliated area and RT-qPCR of *FOXJ1* mRNA. Data normalized to shScramble-transduced control (*n* = 8 donors). (**G**) Top: RNA-seq expression for *SLC25A1 and HIF1A* mRNA in log_10_ (TPM + 0.01) (*n* = 5 donors). Statistical significance was calculated by DESeq2 using the Wald test. Bottom: CiC and HIF1α protein quantification normalized to β-actin in control and *SLC25A1* knockdown cells (*n* = 4 donors). (**H**) Model. Physiological airway O_2_ tension (normoxia) drives the ciliated cell lineage decision in human airway stem cells by stimulating mitochondrial respiration. Mitochondria respiration increases citrate export and stabilizes HIF1α. Both citrate export and HIF1α are required for differentiation into ciliated cells. Bars represent mean [(A), (B), and (G)]. Data represent means ± SEM [(C) and (F)]. *P < 0.05, **P < 0.01, and ***P < 0.001 using repeated measures ANOVA with Dunnett’s multiple comparisons test [(A) and (B)] and paired Student’s *t* test [(C), (F), and (G)].

To address the role of HIF1α, we first examined HIF1α protein levels in basal cells differentiated for 1 day at 0.5, 5, or 18.5% O_2_. As expected, we detected HIF1α protein at 0.5 and 5% O_2_ (Fig. 8A). Unexpectedly, HIF1α protein was also stabilized at 18.5% O_2_, although at lower levels. Given the robust induction of mitochondrial respiration at 18.5% O_2_ (Fig. 2B), we considered that mitochondrial O_2_ consumption might deplete intracellular O_2_ levels and stabilize HIF1α despite the presence of ample extracellular O_2_. In accord, when we blocked oxidative phosphorylation with either electron transport chain (ETC) complex III inhibitor antimycin A or complex V inhibitor oligomycin, HIF1α protein disappeared at 18.5% O_2_ but persisted at 0.5% O_2_ (Fig. 8B).

To test whether HIF1α stabilization is due to increased mitochondrial ROS, we treated cells with S1QEL 1.1 and S3QEL 2, compounds that inhibit ROS generation by complexes I and III, respectively (*37*, *38*). Neither compound decreased HIF1α stabilization at 18.5% O_2_, whereas antimycin A and oligomycin did (fig. S10). These data suggest that HIF1α stabilization at 18.5% O_2_ is unlikely to be due to increased mitochondrial ROS.

HIF1α stabilization at 18.5% O_2_ suggested a potential dual role for HIF1α in basal cells: signaling hypoxia at low environmental O_2_ tension and at higher environmental O_2_ tension signaling mitochondrial respiration to permit ciliated cell differentiation when accompanied by citrate export. To address this, we prevented HIF1α protein accumulation at 18.5% O_2_ by knocking down *HIF1A* (Fig. 8, C and D). *HIF1A* knockdown impaired ciliated cell differentiation (Fig. 8, E and F). Given the apparent cooptation of HIF1α into a mitochondrial respiration-dependent O_2_ response mechanism, we reasoned that *SLC25A1* knockdown would be unlikely to further induce HIF1α. Consistent with this, neither *HIF1A* transcript nor HIF1α protein was induced in *SLC25A1* knockdown cells (Fig. 8G). We deepened our analysis of the *SLC25A1* knockdown-induced hypoxia signature by evaluating differentially accessible ATAC-seq peaks using the same likelihood ratio test as the matching RNA-seq. We queried enrichment of HIF binding motifs in the sequences of differentially accessible regions. In agreement with our data showing that CiC disruption did not further elevate HIF1α levels, the motif for HIF1α was not significantly enriched (*P* = 1). These results suggest that decreased levels of exported citrate signals hypoxia independently of HIF1α protein induction to prevent differentiation into ciliated cells.

## DISCUSSION

Here, we show that when O_2_ tension is relatively high, mitochondrial citrate export licenses the differentiation of airway basal cells into ciliated cells. We also observe an unexpected requirement for mitochondrial respiration-dependent HIF1α stabilization. Together, these findings support a model in which mitochondrial respiration serves as a checkpoint for differentiation into ciliated cells (Fig. 8H).

When an airway stem cell has to decide its fate, O_2_ plays a critical role in determining whether it differentiates into a ciliated cell. Previous studies have shown that basal cells can differentiate into goblet cells both in normoxia and hypoxia (*39*); our data are consistent with that conclusion. However, O_2_ levels covering airway epithelia (levels that would be considered “hyperoxic” in other tissues) are required for differentiation into ciliated cells. Why would “hyperoxia” increase ciliated cell production? Motile cilia are energetically demanding. They are 6- to 7-μm long, each cilium is packed with dynein adenosine triphosphatases that initiate bending, there are 200 to 300 cilia per cell, and they beat continuously 12 to 15 times/s (*40*). We speculate that before committing to the ciliated cell lineage, basal cells sample the environment to ensure that the energy requirements of beating cilia can be met with a continuous supply of ATP that is most efficiently produced when O_2_ powers mitochondrial activity.

Citrate is well-suited as both a readout of energetic competency and an effector of ciliated cell differentiation. First, mitochondrial citrate synthesis requires ETC oxidoreductase activity. Mitochondrial acetyl-CoA production by pyruvate dehydrogenase and β-oxidation, respectively, requires nicotinamide adenine dinucleotide (oxidized form) and flavin adenine dinucleotide regeneration from their reduced forms by ETC oxidation, which in turn requires O_2_. Thus, O_2_ supply from the environment is directly linked to mitochondrial citrate production capacity. Second, because net mitochondrial citrate export requires import of surplus (anaplerotic) fuel substrates into mitochondria, citrate export signals an adequate fuel substrate availability in the cellular milieu (*41*). Third, citrate exported into the cytosol is converted by ACLY into acetyl-CoA, an essential substrate for HATs to regulate the genes controlling ciliated cell differentiation. Cytosolic acetyl-CoA is also a major substrate feeding de novo lipogenesis (*22*, *42*, *43*). This may be important because the costs of building new ciliary membranes are likely high. Thus, citrate exported from mitochondria may serve as both a signal for generation of ciliated cells and building blocks to produce them.

Previous research identified αKG and succinate as key TCA cycle metabolites that signal cellular O₂ availability; αKG and succinate are the substrate and product, respectively, for prolyl-hydroxylases that target HIF1α for degradation (*14*). Under sufficiently hypoxic conditions, mitochondrial NADH accumulates, preventing forward flux to αKG and driving reverse, reductive TCA cycle flux from oxaloacetate to succinate (*44*, *45*). Thus, the succinate to αKG ratio increases. However, hypoxia (0.5% O_2_) did not generate this pattern in airway basal cells; the succinate to αKG ratio fell compared to 18.5% O_2_. These data suggest that airway basal cells may have a uniquely organized O_2_ signaling and response system, perhaps developed because of their exposure to relatively high environmental O_2_ levels. Instead, citrate synthesis may be a more sensitive readout of O_2_ availability.

Our data indicate that mitochondrial citrate export in airway basal cells functions in an O_2_ response mechanism permitting differentiation into ciliated cells. However, we were surprised to find that inhibiting citrate export induced numerous elements of the HIF1α-induced transcriptional program even at 18.5% O_2_. Finding that CiC knockdown phenocopied aspects of a hypoxic transcriptional response led us to discover that HIF1α is required for differentiation into ciliated cells under normoxic conditions. This apparently paradoxical result may use the oxygen responsive capacity of the HIF1α system to enhance metabolic control over differentiation. While HIF1α may be stabilized in response to environmental O_2_ depletion, we speculate that in airway basal cells, HIF1α stabilization in normoxia is coopted to respond to mitochondrial respiration by intracellular O_2_ depletion. This idea aligns with our finding that inhibiting ETC complex III and V both decreased HIF1α stabilization. It is also consistent with previous studies that mitochondrial O_2_ utilization decreases the availability of cytosolic O_2_ (*46*, *47*). Although the complete molecular-biochemical framework regulating differentiation likely comprises many factors, this result supports a two-component model related to mitochondrial respiration; adequate O_2_ supply is required to produce citrate, and sufficiently high mitochondrial respiration is required to deplete intracellular O_2_ to stabilize HIF1α.

Our results illuminate an additional metabolic role for citrate. Previous studies have suggested that citrate can regulate stem cell fate in other tissues. Mesenchymal stem cells reside in a hypoxic (1 to 2% O_2_) bone marrow niche (*48*). In contrast to airway basal cells, mesenchymal stem cells decrease CiC function and histone acetylation at 21% O_2_, preventing their osteogenic differentiation (*48*). Similarly, disrupting CiC prevents differentiation of a trophoblast cell culture model (*49*). Thus, while citrate has a conserved role in regulating stem cell fate, its specific role might partially be determined by O_2_ tension. As O_2_ tensions across tissues vary widely from ~0.5% (large intestine lumen) to ~9% (kidney cortex) (*1*), it is conceivable that stem cells in other tissues with relatively high O_2_ tension may also engage in similar O_2_ response mechanism for their lineage selection.

Finding how mitochondria gate the ciliated cell lineage decision reveals an intriguing link between the airway environment and cellular development with implications for understanding respiratory health and disease. When infection, inflammation, and mucus plug airways, O_2_ tension can plunge to near zero (*50*, *51*). In addition, chronic obstructive pulmonary disease (COPD), asthma, cystic fibrosis, and cigarette smoking can alter mitochondrial morphology and function (*52*–*54*). Our findings raise the possibility that the decreased airway O_2_ tension and mitochondrial damage accrued as the consequences of disease may compromise differentiation of basal cells into ciliated cells, which may introduce a cycle in which the altered airway cell composition further predisposes, in part, to airway disease. For example, the pathologic changes in airway epithelial composition and function associated with COPD persist after smoking cessation (*55*).

This study has advantages and limitations. An advantage is that we used primary cultures of human airway basal cells. When cultured at an ALI, they model in vivo differentiation and allow in-depth metabolic analyses, including ^13^C tracing, that are not possible or less controlled in vivo. A limitation is that we do not address differentiation into rare cell types that may also be affected by O_2_ tension. For example, hypoxia induces differentiation of mouse airway basal cells into solitary neuroendocrine cells (*56*). Another limitation is that by focusing on basal cells in a human model of postnatal differentiation and repair, we have not investigated mechanisms shown in mice by which secretory club cells can transdifferentiate into ciliated cells (*34*). Last, we have not investigated how prenatal differentiation yields airways containing ciliated cells at birth, an interesting problem given that human cord blood delivers O_2_ at ~2.5% (19.3 mm Hg), and levels will be even lower in the fetal lung (*57*). Thus, mechanisms governing ciliated cell differentiation must differ in utero. Consistent with this conclusion, in mouse embryos, airway ciliated cells arise before basal cells from distinct progenitors (*58*).

Overall, these findings identify mechanisms by which the environmental O_2_ in airways promotes differentiation of human airway basal stem cells into ciliated cells. They uncover a central role for mitochondria in airway O_2_ detection and signaling and as a gateway organelle to the ciliated cell lineage decision.

## MATERIALS AND METHODS

### Cell culture

Human airway epithelial cells were isolated from donor tracheal or bronchial tissue as described previously (*7*). Basal cells were selected by brief culture on plastic in BronchiaLife Epithelial Airway Medium (Lifeline). At 80% density, cells were transferred to a transwell membrane (#3470 and #3460, CoStar) at 300,000 cells/cm^2^. After confluence, cells were switched to an ALI by removing apical liquid and fed basolaterally with media containing 50 nM retinoic acid to induce differentiation (*59*). ALI cultures were maintained for 3 weeks at 37°C in 5% CO_2_, with media changed every other day. All studies were approved by the University of Iowa Institutional Review Board. Thirty-three female and 49 male donor lungs were used in this study.

### Lentiviral infection

Basal cells were seeded at 5,500 cells/cm^2^ on plastic. After 24 to 36 hours, cells were infected overnight in polybrene (5 μg/ml) at multiplicity of infection of 2 or 3. For shRNA lentiviruses, puromycin was used at 1 to 1.5 μg/ml for 2 days to select for infected cells. Infected basal cells were transferred to a transwell membrane and differentiated at an ALI for 3 weeks.

### Immunofluorescence

Cells on a transwell membrane were washed once with phosphate-buffered saline (PBS) and fixed with 4% paraformaledehyde for 1 hour at room temperature. Fixed cells were permeabilized with 0.2% Triton X-100 (Thermo Fisher Scientific) in PBS for 20 min and then blocked in Super-Block (Thermo Fisher Scientific) with 5% normal goat serum for 1 hour. Cells were incubated with primary antibodies overnight at 4°C and then with anti-mouse secondary antibody conjugated to Alexa-Fluor 488 (1:1000; Molecular Probes/Invitrogen), anti-rabbit secondary antibody conjugated to Alexa Fluor 568 (1:1000; Molecular Probes/Invitrogen), and phalloidin conjugated to Alexa Fluor 633 (1:1000; Molecular Probes/Invitrogen) for 1 hour at room temperature. The membrane was mounted with VECTASHIELD plus 4′,6-diamidino-2-phenylindole (VectorLabs) and imaged with an Olympus FluoView FV3000 confocal microscope. Primary antibodies used were anti-acetyl-αTUB [1:300; #5335, Cell Signaling Technology (CST)], anti-βTUB IV (1:300; #T7941, BioGenex), anti-MUC5AC (1:5000; #BM5547, OriGene), anti-TP63 (1:400; #13109, CST), and anti-ACSS2 (1:100; #16087-1-AP, Proteintech).

### Immunofluorescence quantification

ImageJ was used to quantify % area covered by acetyl-αTUB–or βTUB IV–positive ciliated cells and MUC5AC-positive goblet cells. Images were opened using the OlympusViewer Plugin. Signal threshold in pixel intensity was set using the “default” automatic thresholding function. The default automatic thresholding function determines the threshold by taking a test threshold and computing the average of the pixels at or below the threshold and pixels above. It then computes the average of those two, increments the threshold, and repeats the process. Incrementing stops when the threshold is larger than the composite average [threshold = (average background + average objects)/2] (*60*). The average of at least five 20× en face images was used per donor. The values were then normalized to the control group.

### Western blotting

Cell lysates were prepared in lysis buffer [1% SDS, 10% glycerol, and 80 mM tris-HCl (pH 6.8)] and boiled for 10 min at 95°C. Proteins were transferred to Immobilon-FL polyvinylidene difluoride membranes (Millipore). Membranes were incubated with primary antibodies overnight at 4°C in 5% bovine serum albumin in tris-buffered saline Tween 20 (TBST) [50 mM tris-HCl, 150 mM NaCl, and 0.05% Tween 20 (pH 8.0)]. After primary antibody binding, membranes were washed three times with TBST and probed with goat anti-rabbit (1:10,000; IRDye 800CW, LI-COR Biosciences) and goat anti-mouse (1:10,000; IRDye 680RD, LI-COR Biosciences) secondary antibodies in TBST for 1 hour at room temperature. After washing three times in TBST, proteins were visualized using an Odyssey Infrared Imaging System (LI-COR Biosciences). Primary antibodies used were anti-TOM70 (1:1000; #14528-1-AP, Proteintech), anti-SLC25A1 (1:1000; #15235-1-AP, Proteintech), anti-HIF1α (1:1000; #36169, CST), anti-βActin (1:5000; #A2228, Sigma-Aldrich), anti-Histone H3 (acetyl K9, K14, K18, K23, K27) (1:1000; #47915, Abcam), and anti-Histone H3 (1:1000; #14269, CST).

### High-resolution respirometry

Oxygen consumption rate (OCR) was measured using the Oroboros Oxygraph-2 k (Oroboros Instruments) (*61*). Briefly, basal cells on a transwell membrane were dissociated with trypsin and resuspended in culture media at 1 million cells/ml. Proton leak was measured after inhibiting the ATP synthase with oligomycin at 1 μM. The maximal OCR was measured after adding FCCP, a protonophore at 1 μM. Residual OCR was measured after inhibiting both the ETC complexes I and III with rotenone at 1 μM and antimycin A at 2 μM, respectively.

### Ussing chamber experiments

Airway epithelia on a transwell membrane were mounted in modified Ussing chambers (Physiologic Instruments) and bathed in Krebs-Ringer buffer solution containing 118.9 mM NaCl, 25 mM, NaHCO_3_, 2.4 mM K_2_HPO_4_, 0.6 mM KH_2_PO_4_, 1.2 mM MgCl_2_, 1.2 mM CaCl_2_, and 5 mM dextrose at 37°C and adjusted to pH 7.4 in the presence of 5% CO_2_. Apical and basolateral chambers were voltage clamped, followed by recording of short-circuit current (*I*_SC_) and transepithelial conductance (*G*_t_) at baseline and in response to the sequential apical addition of 100 μM amiloride, 100 μM 4,4′-diisothiocyano-2,2′-stilbenedisulfonic acid, 10 μM forskolin, and 100 μM CFTR_inh_-172.

### Metabolite extraction

Cells on a transwell membrane were washed twice with ice-cold PBS, twice with ice-cold water before flash freezing in liquid nitrogen. Cells were lyophilized overnight then resuspended in 1 ml of ice-cold 2:2:1 methanol/acetonitrile/water containing a pool of internal standards for gas chromatography–mass spectrometry (GC-MS) profiling (D_4_-citric acid, D_4_-succinic acid, D_8_-valine, and [U]^13^C-labeled glutamine, glutamic acid, lysine, methionine, serine, and tryptophan; Cambridge Isotope Laboratories) or D_8_-valine alone for liquid chromatography MS (LC-MS) ^13^C-tracing to extract metabolites. The mixtures were transferred to a microcentrifuge tube and flash-frozen in liquid nitrogen, thawed for 10 min in a water bath sonicator, and rotated for 1 hour at −20°C. Mixtures were centrifuged for 10 min at 21,000*g*, and 300 or 200 μl of the cleared metabolite extracts were transferred to autosampler vials or microcentrifuge tubes for GC-MS or LC-MS, respectively, and were dried using a SpeedVac vacuum concentrator (Thermo Fisher Scientific). For GC-MS, dried metabolite extracts were reconstituted in 20 μl of methoxyamine (11.4 mg/ml) in anhydrous pyridine, vortexed for 5 min, and heated for 1 hour at 60°C. Next, 16 μl of *N*,*O*-bis(trimethylsilyl) trifluoroacetamide was added to each sample, and samples were vortexed for 1 min and heated for 30 min at 60°C. Derivatized samples were analyzed by GC-MS. A total of 1 μl of derivatized sample was injected into a Trace 1300 GC (Thermo Fisher Scientific) fitted with a TraceGold TG-5SilMS column (Thermo Fisher Scientific). For LC-MS, dried extracts were reconstituted in 20 μl of acetonitrile/water (1:1 v/v) vortexed well, rotated on a rotator in −20 °C overnight, centrifuged and the supernatant was transferred to LC-MS autosampler vials for analysis. LC-MS data were acquired on a Thermo Q Exactive hybrid quadrupole Orbitrap mass spectrometer with a Vanquish Flex UHPLC system or Vanquish Horizon UHPLC system. The LC column used was a Millipore SeQuant ZIC-pHILIC (2.1 mm by 150 mm, 5-μm particle size) with a ZIC-pHILIC guard column (20 mm by 2.1 mm). The injection volume was 2 to 5 μl. The mobile phase comprised buffer A [20 mM (NH_4_)_2_CO_3_, 0.1% NH_4_OH (v/v)] and buffer B [acetonitrile]. The chromatographic gradient was run at a flow rate of 0.150 ml/min as follows: 0- to 21-min linear gradient from 80 to 20% buffer B; 20- to 20.5-min linear gradient from 20 to 80% buffer B; and 20.5- to 28-min hold at 80% buffer B. The MS operated in positive or negative timed single ion monitoring mode with the spray voltage set to 3.0 kV, the heated capillary held at 275°C, and the Heated Electrospray Ionization (HESI) probe held at 350°C. The sheath gas flow was set to 40 units, the auxiliary gas flow was set to 15 units, and the sweep gas flow was set to 1 unit. MS data resolution was set at 70,000, the Automatic Gain Control (AGC) target at 10 × 10^6^, and a maximum injection time of 200 ms.

### ^13^C tracing

Cells on a transwell membrane were incubated with media containing 3 mM unlabeled and 12.5 mM [U]-^13^C_6_–labeled glucose (Cambridge Isotope Laboratories) for 5 hours. Cells were washed twice with ice-cold PBS and twice with ice-cold water, then snap-frozen in liquid nitrogen, and processed for metabolite extraction and LC-MS analysis.

### GC-MS/LC-MS data analysis

Raw data were analyzed using TraceFinder 5.1 (Thermo Fisher Scientific). Targeted metabolites were identified based on the University of Iowa Metabolomics Core facility standard-confirmed, in-house library defining a target ion and at least one confirming ion and retention time (GC) or accurate mass, retention time, and MS/MS fragmentation pattern when present (LC). For metabolomic profiling, a pooled-sample was analyzed at the beginning, at a set interval during, and at the end the analytical run to correct for instrument drift using the NORmalization and EVAluation of Metabolomics Data (NOREVA) tool (*62*). NOREVA-corrected individual metabolite values (arbitrary units) were then normalized: first, per sample, to the sum of identified metabolite values to control for extraction, derivatization, and/or loading effects; and second, across samples, dividing individual metabolite values by the average of that metabolite in a reference condition to set the reference condition to 1 while preserving error and to render metabolite values by fold of the reference. For ^13^C-tracing analysis, ^12^C-natural abundance was corrected using the isotopolog distributions generated from samples not treated with tracer and the previously defined equations (*63*).

### Quantitative Real-time PCR

RNA was extracted using a RNeasy Mini Kit or RNeasy Micro Kit (QIAGEN) and treated with deoxyribonuclease I (DNase I) (QIAGEN) as per the manufacturer’s instructions. cDNA was prepared using a High-Capacity cDNA Reverse Transcriptase kit (Applied Biosystems). Quantitative real-time polymerase chain reaction (qRT-PCR) was performed on QuantStudio 6 Flex Real-Time PCR System (Applied Biosystems) using Fast SYBR Green Master Mix (Applied Biosystems). Gene expression was quantified by first subtracting the Threshold Cycle (Ct) of *TBP* encoding the TATA-Box Binding Protein (control for cDNA input) from the Ct of the gene of interest (ΔCT) followed by subtracting the average ΔCT of the control group from the ΔCT of each sample. The resulting value was multiplied by −1 (−ΔΔCT).

### Chromatin immunoprecipitation–qPCR

Chromatin immunoprecipitation (ChIP) was performed using ChIP-IT High Sensitivity kit from Active Motif. Briefly, basal cells were harvested from transwell membranes by trypsinization and cross-linked with 1% formaldehyde for 10 min at room temperature. After cell lysis, chromatin was fragmented to approximately 200 to 1200 base pairs in length by sonicating for 30 cycles of 30 s on/off for a total sonication “on” time of 15 min. Cell lysates were incubated with 4 μg of anti-H3K27Ac (#ab4729, Abcam) or immunoglobulin G control (#2729S, CST) at 4°C overnight. ChIP DNA was eluted in 100 μl of elution buffer and 4 μl was used for qPCR.

### RNA-seq analysis

RNA was extracted using a RNeasy Mini Kit (QIAGEN) as per the manufacturer’s instructions. After digestion of genomic DNA with DNase I (QIAGEN), RNA quality was assessed using an Agilent BioAnalyzer 2100 (Agilent Technologies). Ribosomal RNA depletion and sample library preparation were done using the Illumina TruSeq Stranded Total RNA with RiboZero. Barcoded samples were pooled and sequenced using an Illumina NovaSeq 6000 in the Iowa Institute of Human Genetics (IIHG) Genomics Core Facility. Paired-end reads were demultiplexed and converted from the native Illumina BCL format to fastq format using an in-house python wrapper to Illumina’s ‘bcl2fastq’ conversion utility. Fastq data were processed with nf-core/rnaseq (version 3.6), a best practices pipeline available at the open-source “nf-core” project (https://nf-co.re/, Nextflow version 22.04). Reads from the samples were aligned against the Ensembl genomic reference “GRCh37” using the STAR aligner. Concurrently, reads were also pseudo-aligned to the transcriptome using Salmon (*64*). Salmon performs its own internal quantitation, yielding estimated counts and values in length-normalized TPM (transcripts per million). Transcript-level abundances were then converted to gene-level counts. Quality control was performed with samtools and qualimap, a computational tool that detects common QC problems (*65*–*68*). Gene-level counts were used for differential gene expression (DE) analysis with R/DESeq2 (*69*). The Bioconductor package “PCAExplorer” was used for exploratory analysis (*70*). Advaita Bio’s iPathwayGuide (www.advaitabio.com/ipathwayguide) was used to perform pathway analysis on the DE gene lists. This software analysis tool implements the “Impact Analysis” approach that takes into consideration the direction and type of all signals on a pathway (*35*, *36*, *71*, *72*). The raw fastq files and associated metadata have been made available for download at NCBI-GEO (accession GSE282141).

### ATAC-seq analysis

ATAC-seq libraries were generated using an ATAC-seq kit from Active Motif. Briefly, cells on a transwell membrane were washed once with ice cold PBS. ATAC lysis buffer was added directly to the cells and incubated for 10 min. Cells were scraped off and nuclei were collected by spinning at 500*g* for 5 min at 4°C. Subsequent steps were performed according to the manufacturer’s instructions. The library quality and fragment size distribution were analyzed by an Agilent BioAnalyzer 2100 (Agilent Technologies). Sequencing (150-bp paired end reads) was performed using the Illumina NovaSeq 6000 sequencer in the IIHG Genomics Core Facility. The ATAC-Seq data were analyzed using a custom computational pipeline developed with Nextflow (www.nextflow.io). FastQC (version 0.11.5) was used to assess quality of the reads, and ngmerge (version 0.3) was used for read trimming. Trimmed reads were aligned to the human genome (reference GRCh37) using the bwa mem aligner (version 0.7.15-r1140). Peak calling was performed with Genrich (version 0.5) using the following filtering options: removal of PCR duplicates and retention of unpaired alignments. The default *P* value of 0.01 was used for statistical significance. Processed ATAC-seq data containing peak location information (narrowPeak format) were imported into R (version 4.0.0) and analyzed using the ChIPseeker package (version 1.18.0). Association of peaks with genes was performed by the ChIPseeker annotatePeak function using gene location data from the UCSC known genes database. For differential occupancy analysis of the ATAC-Seq peaks, the diffBind package (version 3.0.15) was used to generate counts data for each peak location and R/DESeq2 was used to compute statistical metrics. To identify enriched transcription factor binding motifs in the differential peaks, we first extracted peak sequences using the Biostrings package (version 2.50.2) in R. The resulting fasta files were then analyzed with the Homer program filterMotifs.pl (version 4.11.1) using default parameters. The raw fastq files and associated metadata have been made available for download at NCBI-GEO (accession GSE282650).

### Plasmids

Bacterial glycerol stock for all shRNA viral plasmids were purchased from Sigma-Aldrich except for the scramble shRNA (#1864, Addgene). Human wild-type *SLC25A1* cDNA (#41904, DNAsu repository) was used as a template to generate the p.R282H mutant using the gene splicing by overlap extension method (*73*). The codon CGC coding for arginine at the amino acid position 282 in wild-type cDNA was mutated to CAC coding for histidine using the primers SLC25A1 (R282H)-FWD (5′-GGCACTGTCCCCCACCTGGGCCGGGTC-3′) and SLC25A1 (R282H)-RVS (5′-GACCCGGCCCAGGTGGGGGACAGTGCC-3′). cDNAs encoding wild-type *SCL25A1* and p.R282H mutant, codon-optimized *SLC13A5* (#161111, Addgene), and *ACSS2* (#OHu18043, GenScript) were cloned into the pMA1 lentiviral vector (*74*, *75*).

### Study design and analysis

Each experiment was replicated at least twice using different donors. No data were excluded from the analysis.

### Statistical analysis

Prism v10.2.3 (GraphPad) was used to generate graphs and perform statistical analyses as indicated in the text and figure legends. All Student’s *t* test results reported here are two tailed.
