# Supplementary material for: The oxygen level in air directs airway epithelial cell differentiation by controlling mitochondrial citrate export
Source: Sci Adv. 2025 Jan 24;11(4):eadr2282. doi: 10.1126/sciadv.adr2282 (PMC11759043; doi:10.1126/sciadv.adr2282)
Supplement: Supplementary file 1 — Figs. S1 to S10 Table S1 Legends for data S1 and S2 [file sciadv.adr2282_sm.pdf]

Science Advances
AAAS

Supplementary Materials for

The oxygen level in air directs airway epithelial cell differentiation by controlling mitochondrial citrate export

Bo Ram Kim *et al.*

Corresponding author: Eric B. Taylor, eric-taylor@uiowa.edu; Michael J. Welsh, michael-welsh@uiowa.edu

Sci. Adv. **11**, eadr2282 (2025)
DOI: 10.1126/sciadv.adr2282

The PDF file includes:

Figs. S1 to S10
Table S1
Legends for data S1 and S2

Other Supplementary Material for this manuscript includes the following:

Data S1 and S2

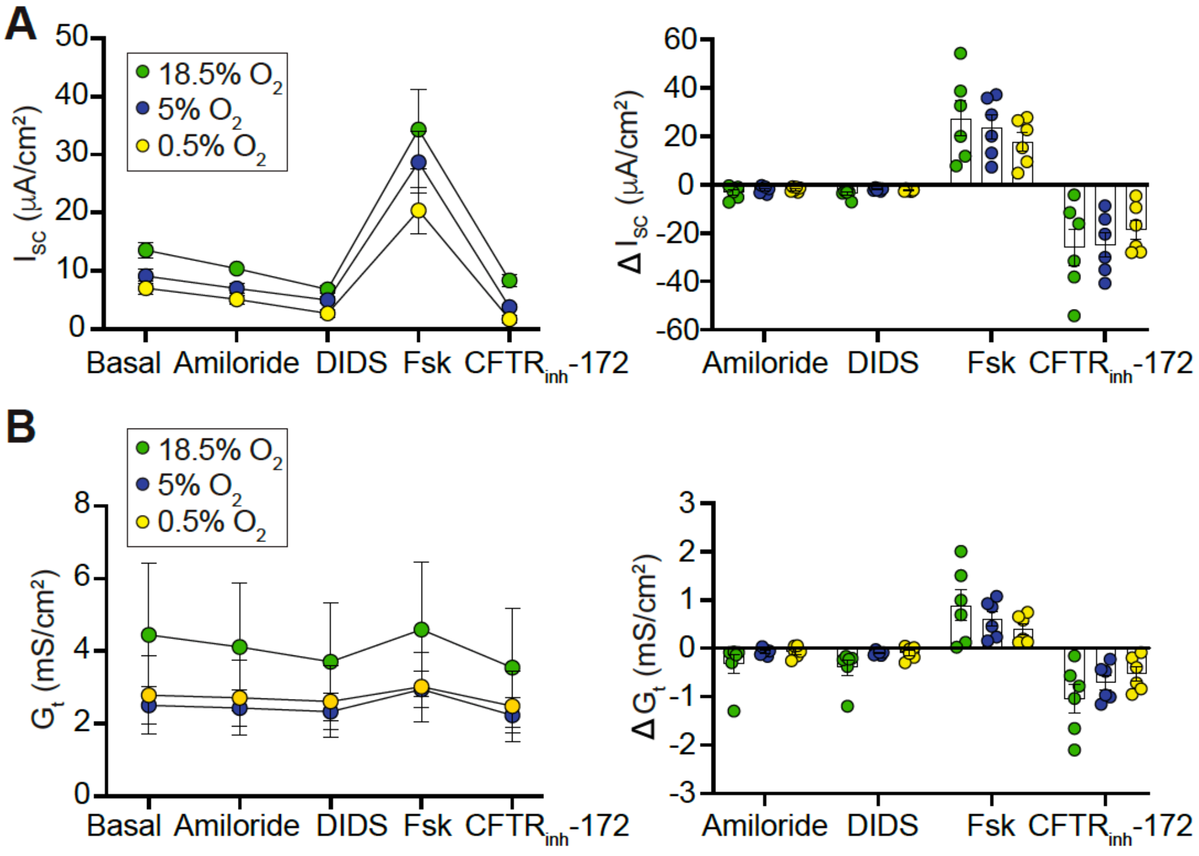

Fig. S1. Airway epithelia differentiated at different O_2 tensions exhibit typical electrophysiological properties.

(**A, B**) Airway epithelia differentiated at 18.5%, 5%, and 0.5% O_2 are placed in Ussing chambers with symmetric Krebs-HCO_3-solution gassed with 5% CO_2. Epithelia were voltage-clamped, and short-circuit current (I_{sc}) (A) and transepithelial conductance (G_t) (B) were recorded as pharmacologic agents were sequentially added to the apical chamber (n=6). Concentrations: 100 µM amiloride, 100 µM DIDS, 10 µM forskolin, and 100 µM CFTR$_{inh}$-172. Abbreviations: DIDS, 4,4′-Diisothiocyano-2,2′-stilbenedisulfonic acid; Fsk, Forskolin. Data represent mean ± SEM. No statistical significance was found using repeated measures ANOVA with Tukey's multiple comparisons test.

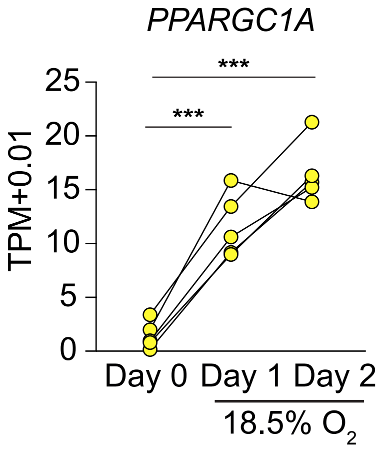

Fig. S2. *PPARGC1A* is induced in differentiating basal cells upon exposure to 18.5% O_2. RNAseq expression values for *PPARGC1A* in log10 (TPM+0.01) at day 0 (pre-differentiation), day 1, and day 2 post-differentiation at 18.5% O_2 (n=5 donors). ****P* < 0.001 using repeated measures ANOVA with Dunnett's multiple comparisons test.

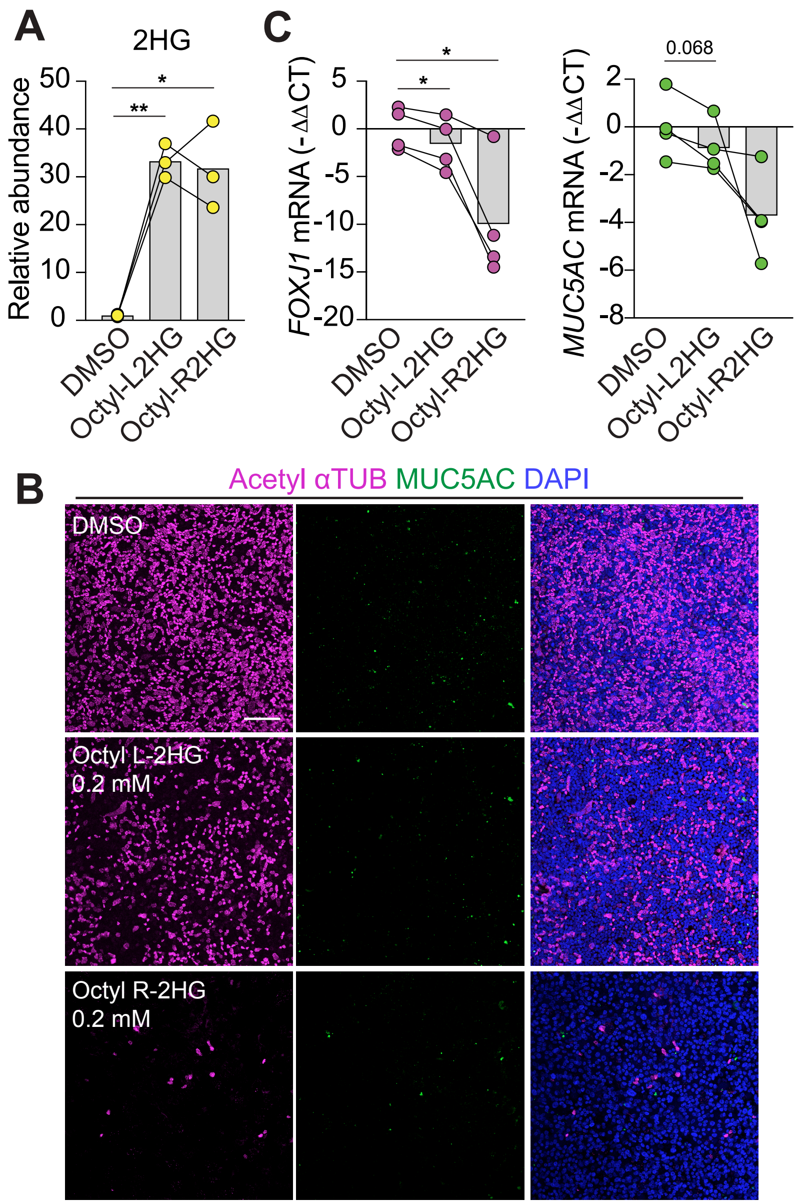

Fig. S3. 2HG inhibits basal cell differentiation into both goblet and ciliated cells.
(**A**) Relative abundance of 2HG in basal cells 1 hour after treatment with 0.2 mM of octyl L- and octyl R-2HG (n=3 donors). (**B, C**) Basal cells were treated with 0.2 mM of octyl L- or octyl R-2HG during differentiation at an ALI. (**B**) *En face* images of ciliated cells expressing acetyl-αTUB (magenta) and goblet cells expressing MUC5AC (green). DAPI is blue. Scale bar: 100 µm. (**C**) RT-qPCR analysis of *FOXJ1* and *MUC5AC* mRNA in differentiated epithelia (n=4 donors). Data are normalized to DMSO control. Bars represent mean. *$P < 0.05$, **$P < 0.01$, repeated measures ANOVA with Dunnett's multiple comparisons test (A, C).

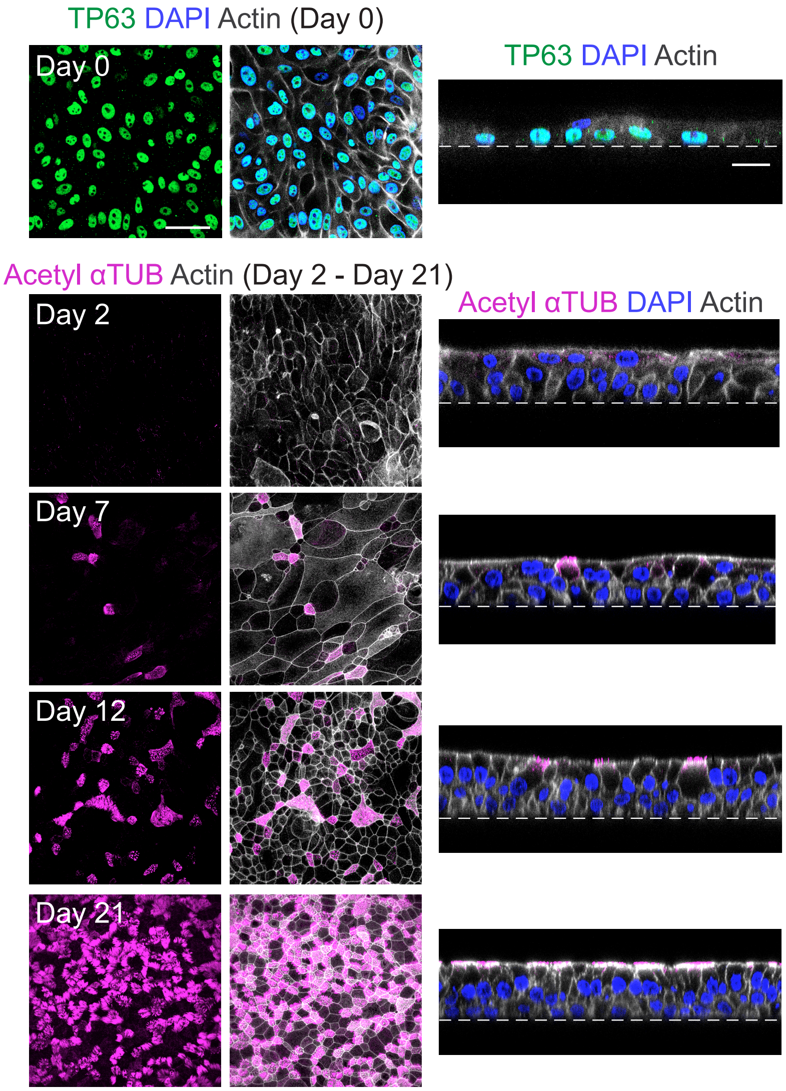

Fig. S4. Timecourse analysis of differentiation of airway basal cells into ciliated cells at 18.5% O_2.

En face and cross section images tracking ciliated cell differentiation at day 0, 2, 7, 12, and 21. TP63, basal cell marker; acetyl-αTUB, ciliated cell marker. Scale bars: 50 µm (*en face*); 20 µm (cross section).

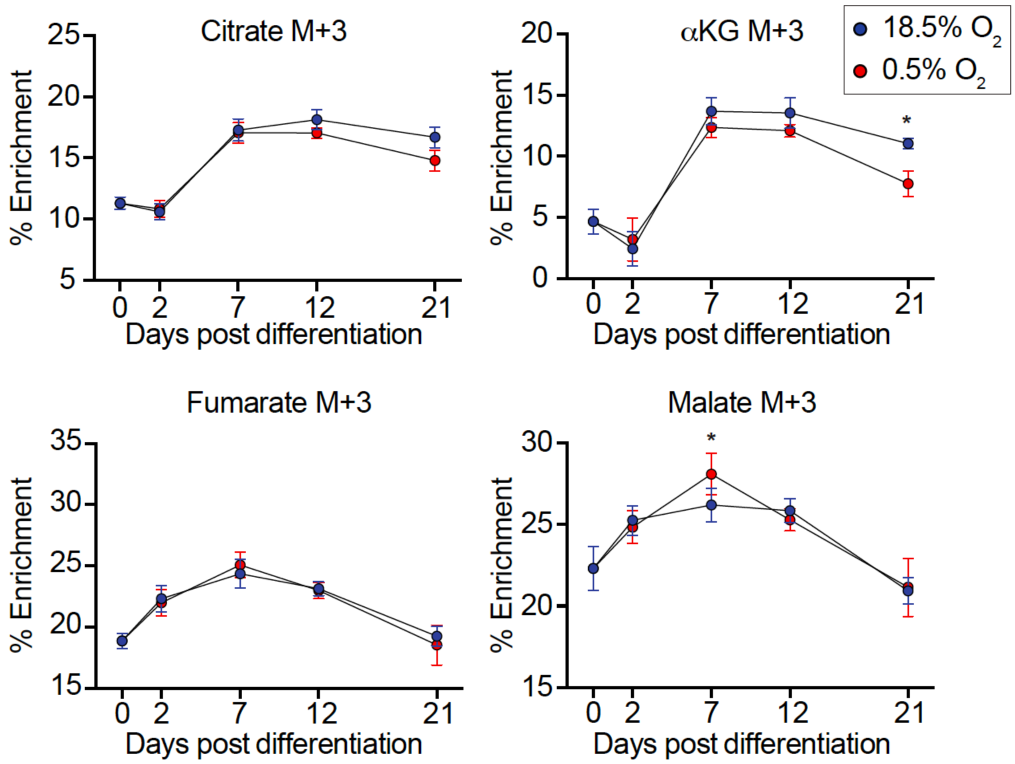

Fig. S5. % M+3 ^{13}C enrichment patterns of major TCA cycle metabolites during airway epithelial differentiation at 18.5% and 0.5% O_2.
Changes in % M+3 ^{13}C isotopologues of citrate, αKG, fumarate, and malate during differentiation at 18.5% O_2 (blue circle) and 0.5% O_2 (red circle) (n=6 donors). Data represent mean ± SEM. *$P < 0.05$ using paired Student's t test.

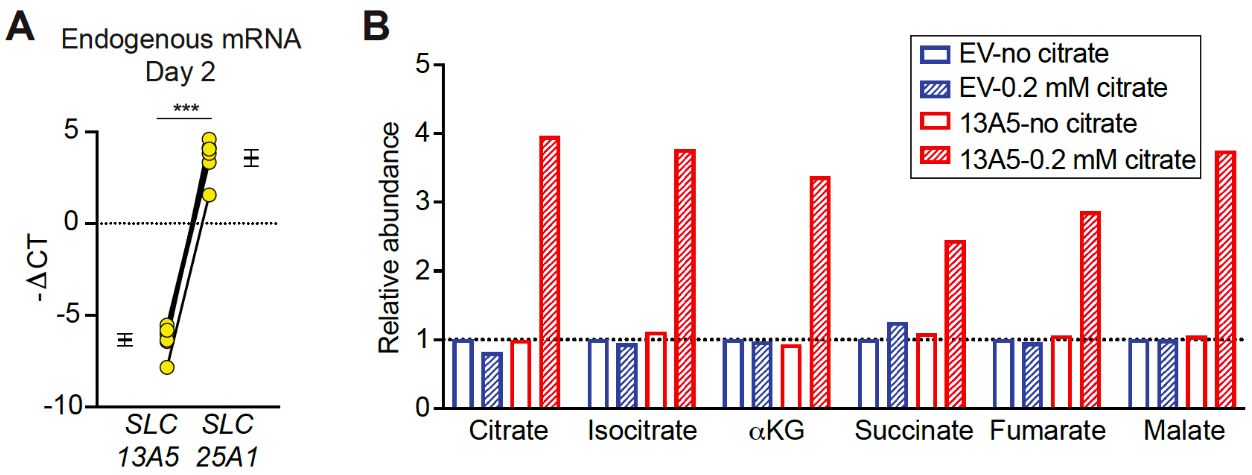

Fig. S6. Ectopic *SLC13A5* expression in airway basal cells facilitates uptake of supplemented citrate.
(**A**) RT-qPCR analysis of endogenous *SLC13A5* and *SLC25A1* mRNA at ALI day 2. The y-axis represents the -ΔCT between *SLC13A5* or *SLC25A1* and *TBP* (TATA-Box Binding Protein; control for cDNA input) (n=6 donors). (**B**) Relative abundance of TCA cycle metabolites in lenti-empty vector (EV) and lenti-SLC13A5 (13A5)-transduced basal cells at ALI day 1. Cells were supplemented with and without 0.2 mM citrate. Data are normalized to EV-transduced control without citrate supplementation (n=1 donor). Data represent mean ± SEM (a). ***$P < 0.0001$ using paired Student's t test (a).

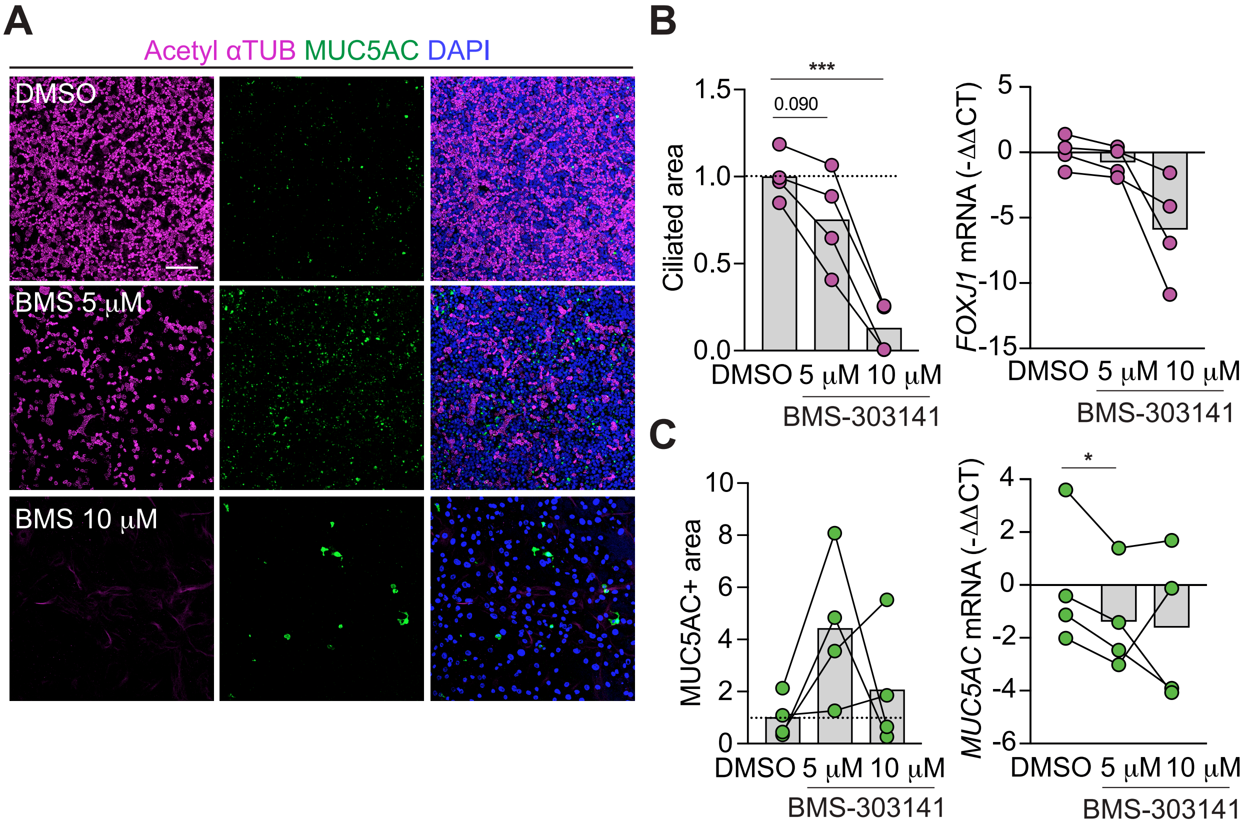

Fig. S7. ACLY inhibition decreases ciliated cell differentiation.
(**A-C**) Basal cells were treated with either DMSO (vehicle control) or ACLY inhibitor BMS-303141 at 5 μM and 10 μM during differentiation. (**A**) *En face* images of ciliated cells expressing acetyl-αTUB (magenta) and goblet cells expressing MUC5AC (green). Scale bars: 100 μm. (**B**) % Ciliated area and RT-qPCR analysis of *FOXJ1* mRNA (n=4 donors). (**C**) % MUC5AC-positive area and RT-qPCR analysis of *MUC5AC* mRNA (n=4 donors). Data are normalized to DMSO-treated control. Bars represent mean. **P* <0.05, ****P* < 0.001 using repeated measures ANOVA with Dunnett's multiple comparisons test (B, C).

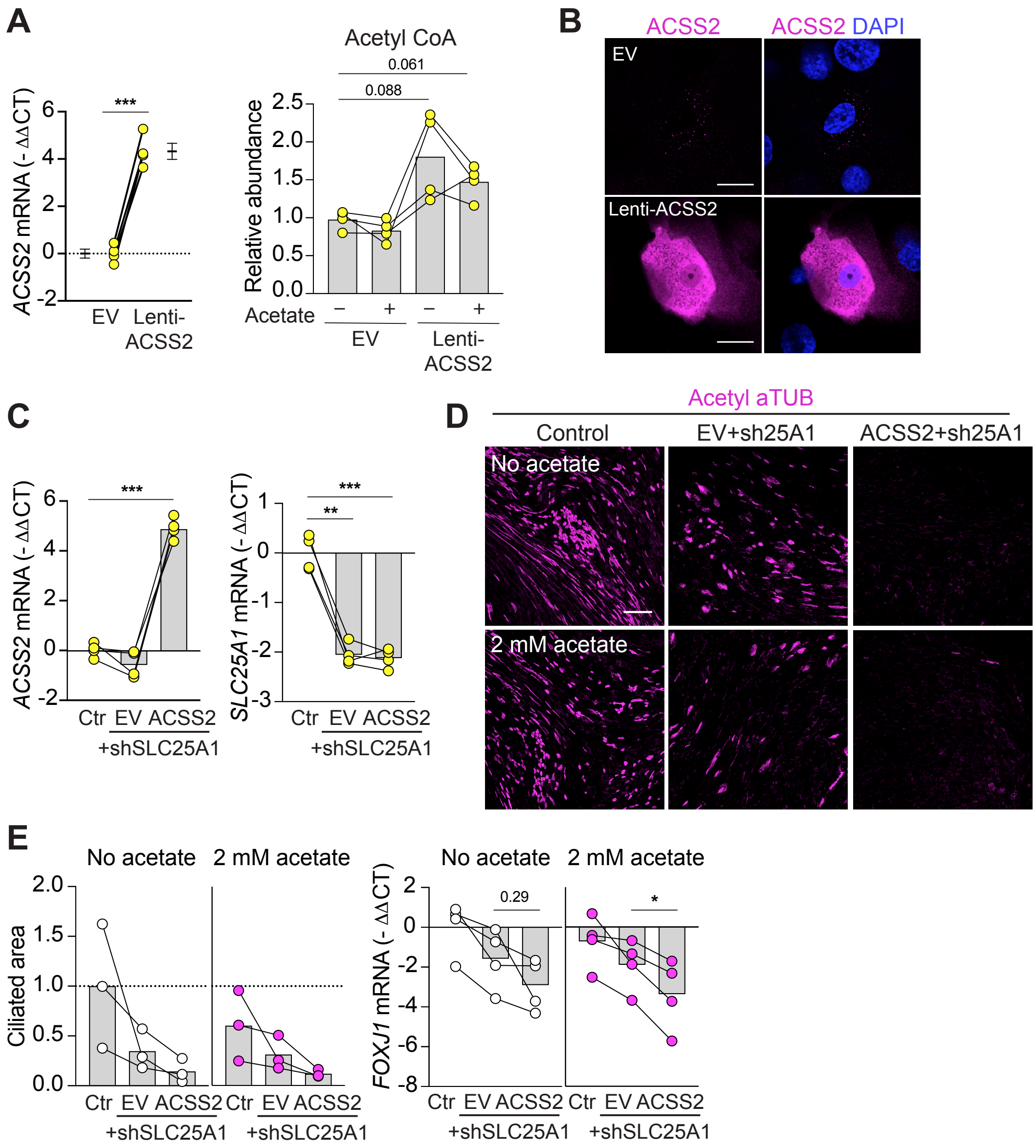

Fig. S8. ACSS2 overexpression with acetate supplementation does not rescue ciliated cell differentiation in CiC KD basal cells.

(**A, B**) Basal cells were transduced with lenti-empty vector (EV) or lenti-ACSS2. (**A**) RT-qPCR analysis of *ACSS2* mRNA and acetyl-CoA abundance with and without 2 mM of acetate at ALI day 2. Data are normalized to EV without acetate (n=4 donors). (**B**) Single slice 2D images of ACSS2 (magenta) in EV- and lenti-ACSS2-transduced basal cells. Scale bars: 20 µm. (**C-E**) Control group (Ctr) was co-infected with shScramble and lenti-empty vector (EV) viruses. Other groups were co-infected with shSLC25A1 and lenti-EV or lenti-ACSS2 viruses. (**C**) RT-qPCR analysis of *ACSS2* and *SLC25A1* mRNA at ALI day 2. Data are normalized to the control group (n=4 donors) (**D**) *En face* images of ciliated cells expressing acetyl-αTUB (magenta). Scale bar: 100 µm. (**E**) % Ciliated area and RT-qPCR analysis of *FOXJ1* mRNA in differentiated epithelia. Data are normalized to the control group (n=3 donors for ciliated area; n=4 donors for RT-qPCR). Data represent mean ± SEM (A). Bars represent mean (A, C, E). *$P < 0.05$, **$P < 0.01$, ***$P < 0.001$ using paired Student's t test (A) and repeated measures ANOVA with Dunnett's multiple comparisons test (A, C, E).

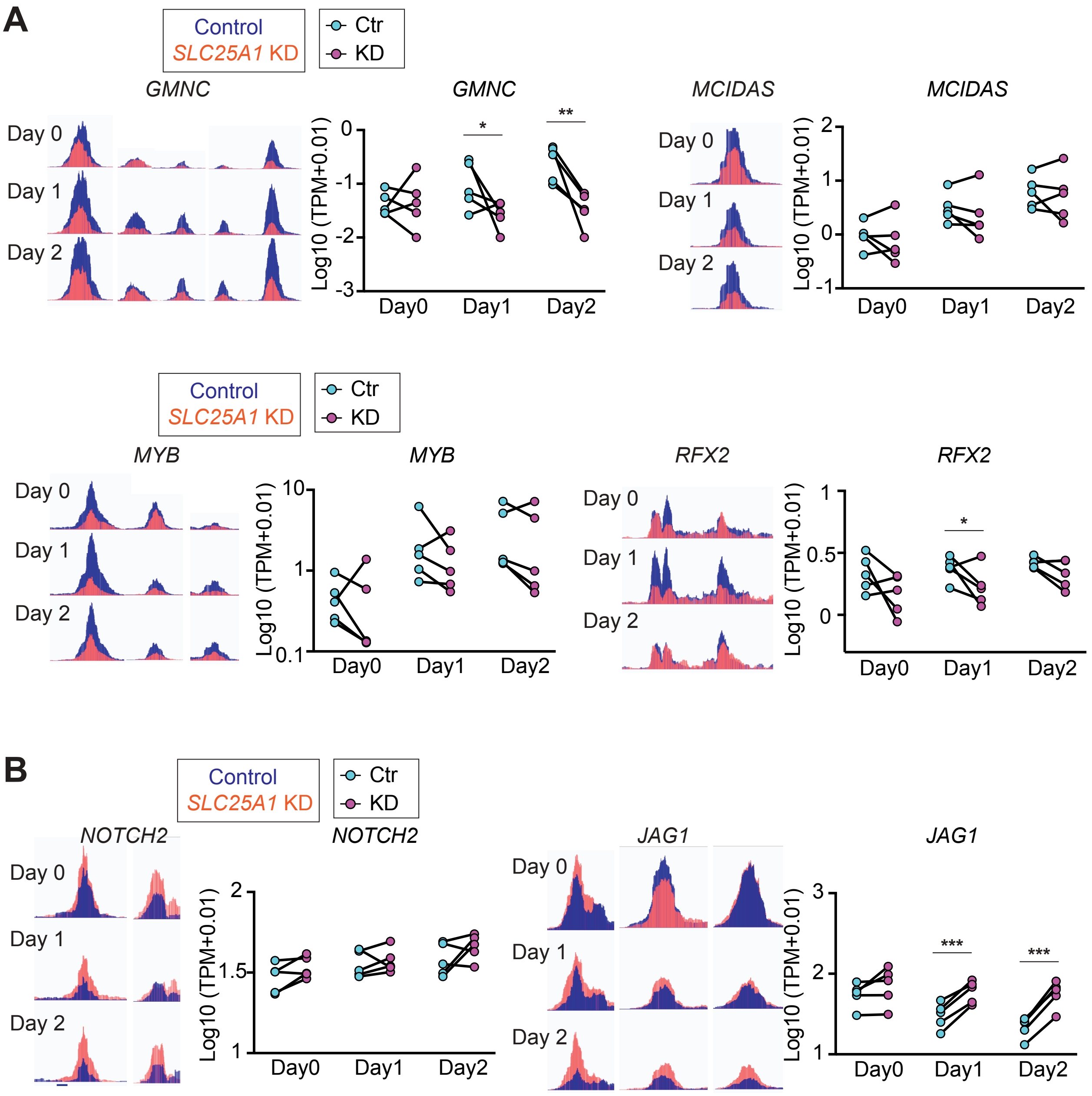

A
Control
SLC25A1 KD
Ctr
KD
GMNC
GMNC
MCIDAS
MCIDAS
Day 0
Day 1
Day 2
Log10 (TPM+0.01)
Day0
Day1
Day2
MYB
MYB
RFX2
RFX2
B
NOTCH2
NOTCH2
JAG1
JAG1

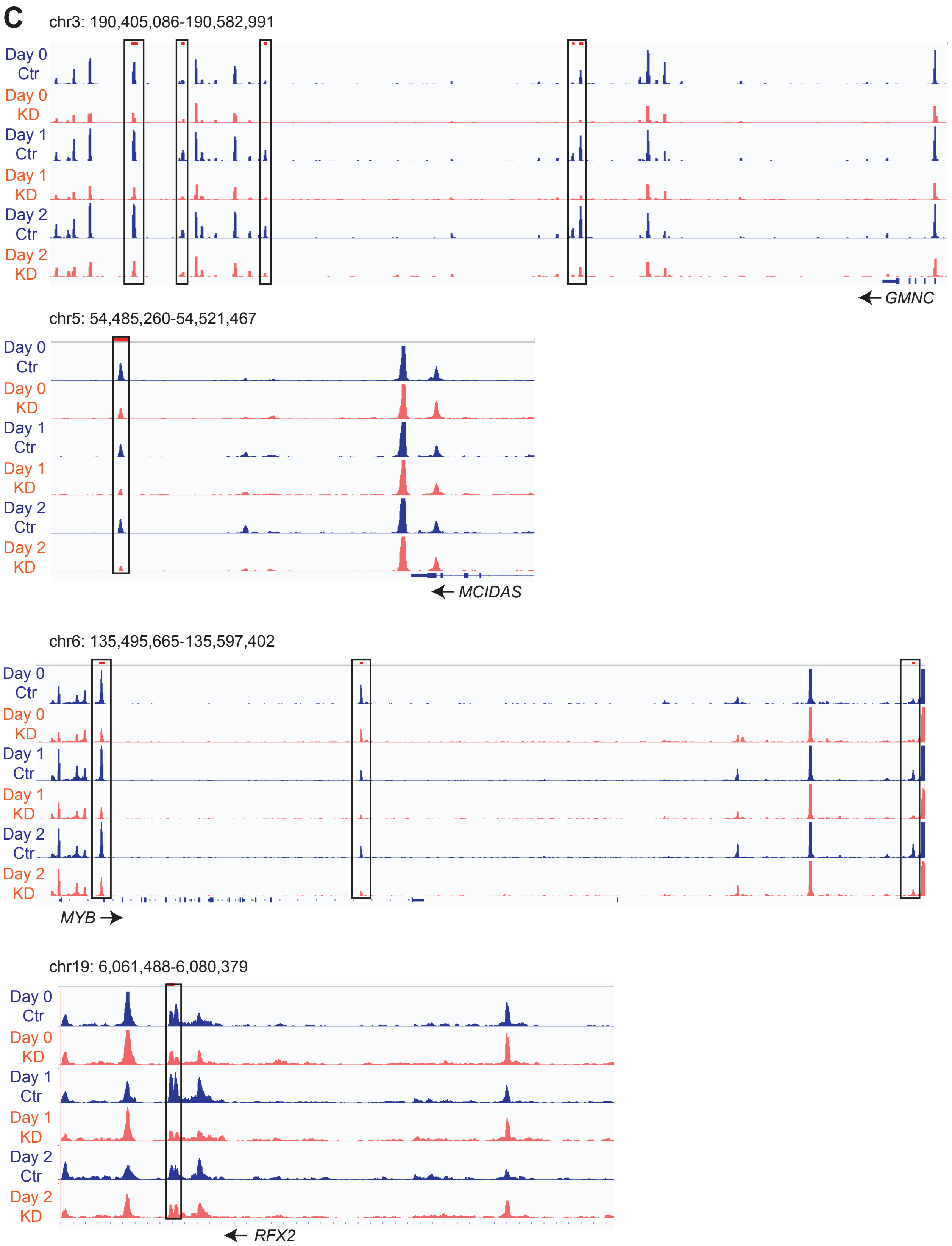
C
chr3: 190,405,086-190,582,991
Day 0 Ctr
Day 0 KD
Day 1 Ctr
Day 1 KD
Day 2 Ctr
Day 2 KD
GMNC
chr5: 54,485,260-54,521,467
Day 0 Ctr
Day 0 KD
Day 1 Ctr
Day 1 KD
Day 2 Ctr
Day 2 KD
MCIDAS
chr6: 135,495,665-135,597,402
Day 0 Ctr
Day 0 KD
Day 1 Ctr
Day 1 KD
Day 2 Ctr
Day 2 KD
MYB
chr19: 6,061,488-6,080,379
Day 0 Ctr
Day 0 KD
Day 1 Ctr
Day 1 KD
Day 2 Ctr
Day 2 KD
RFX2

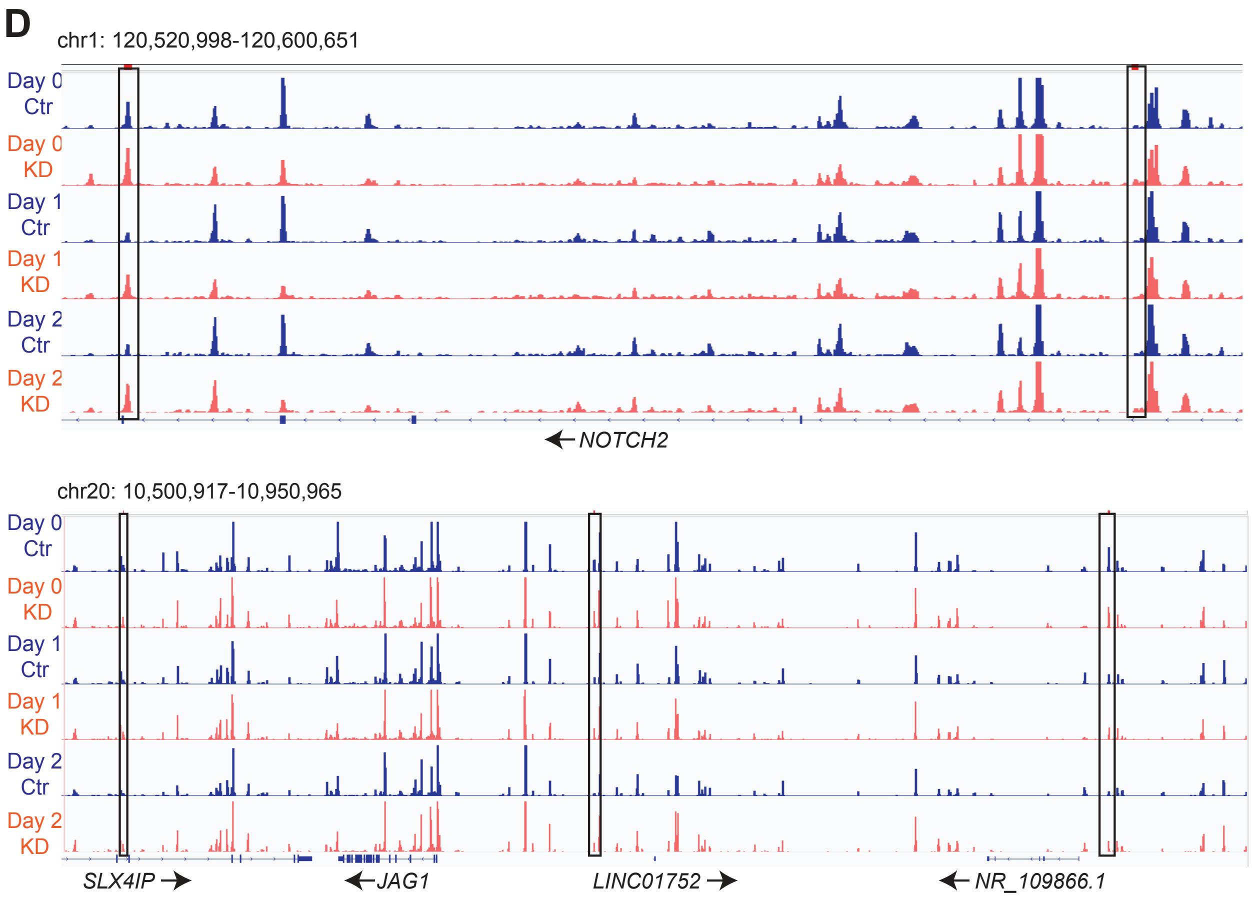

Fig. S9. Citrate export influences chromatin accessibility and transcriptional activation of ciliated cell lineage genes.
(**A, B**). Overlay of differentially accessible ATAC-seq peaks ($P < 0.05$) between control (Ctr) and *SLC25A1* knockdown cells (KD). Each peak represents the average of 5 donors. To the right of the ATAC-seq peaks, RNAseq values for the indicated genes are shown in log10 (TPM+0.01) (n=5 donors). **P* <0.05, ***P* < 0.01, ****P* < 0.001. Statistical significance was calculated by DESeq2 using the Wald test. (**C, D**) Normalized ATAC-seq sequencing tracks of control and *SLC25A1* KD cells at day 0 (pre-differentiation), and day 1 and day 2 post-differentiation. Each track represents the average of 5 donors. Differentially accessible regions ($P < 0.05$) at day 1 and/or day 2 are shown in box. Arrows point to the direction of transcription.

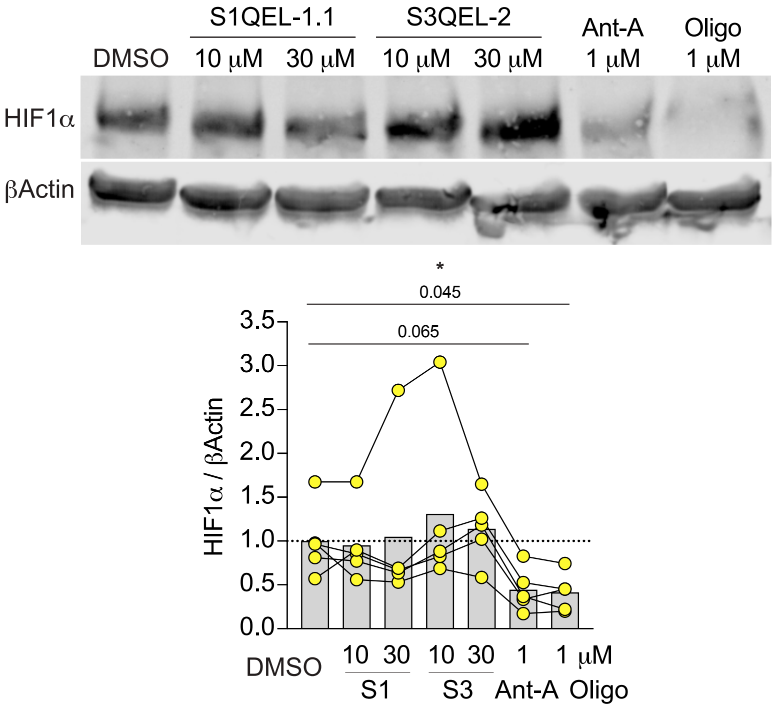

Fig. S10. Blocking mitochondrial ROS does not abolish HIF1α stabilization at 18.5% O_2. (Top) Western blot of HIF1α protein in basal cells differentiated for one day at 18.5% O_2. Cells were treated overnight with DMSO vehicle control, 10 or 30 μM of S1QEL 1.1 (S1) and S3QEL 2 (S3), 1 μM antimycin A (Ant A), and 1 μM of oligomycin (Oligo). (Bottom) The graph shows quantification of HIF1α protein normalized to βActin (n=5 donors). Bars represent mean. *$P <$ 0.05 using repeated measures ANOVA with Dunnett's multiple comparisons test.

Table S1. qPCR primer sequences

Gene	Forward Primer	Reverse Primer
FOXJ1	CCCACCTGGCAGAATTCAATCCG	CTCAGTAGCCGCTCCGCGTAC
MUC5AC	TACCAGAACCAGTCGACCTGTGC	ACCGTGTTGCCCTCGAGCGAGTAC
SLC25A1	TCAAGGCATTCTACAAGGGCA	TTCCACACTTTGTTGAGCAGC
ACLY	GGCTAACACCCCTTCAGTCC	GCAGGTAGCAGAGCAAAGTC
HIF1A	GCTCCCTATATCCCAATGGA	GCTTGCGGAACTGCTTTC
PPARGC1A	GACCCCAGAGTCACCAAATGA	GGCCTGCAGTTCCAGAGAGT
TBP	TGTGCACAGGAGCCAAGAGT	ATTTTCTTGCTGCCAGTCTGG
SLC13A5 (Endogenous)	GGAAGAGCCACAAGACCACA	CGTTGGGTCATTTTGGGGTG
SLC13A5 (Codon optimized)	TGGTGGCCGTGTTTACAGAG	GATAGACCGGGACATGCTGG
ACSS2	CAAGAGGCAGGGGATGAGTG	ATGTAGCCCCCAACTGTGTG
FOXJ1-Promoter-1	AAGAGAGTTGCCGCCAGG	CGCCCCCATAAACAGCTTCC
FOXJ1-Promoter-2	CTTCCCGCCCCTTTCATAGTT	CCTGTCACCTGCTGCTTCT
FOXJ1-Promoter-3	ACCTGCTTCTCTCCGTTCTC	GAAGGAGCAGGATGCGTGTC

Data S1. (separate file)

Metabolomics data for airway basal cells prior to differentiation (day 0) and one day post-differentiation at 18.5% O_2 and 0.5% O_2

Data S2. (separate file)

^{13}C-enrichment data for major TCA cycle metabolites during airway epithelial differentiation at 18.5% and 0.5% O_2
